# Comparative and Histology-Aware Bioinformatic Characterization of FAM171A1 and FAM171A2 in Sarcoma: Expression, Prognostic, and Immune Associations

**DOI:** 10.3390/biomedicines14102362

**Published:** 2026-10-16

**Authors:** Gonul Seyda Seydel, Durmus Ayan

**Affiliations:** 1 Health Care Services, Nigde Omer Halisdemir University, Nigde 51240, Türkiye; seydaseydel@hotmail.com; 2 Medical Biochemistry, Faculty of Medicine, Nigde Omer Halisdemir University, Nigde 51240, Türkiye; 3 Medical Biochemistry, Dr. Abdurrahman Yurtaslan Ankara Oncology Training and Research Hospital, Ankara 06200, Türkiye

**Keywords:** sarcoma, FAM171A1, FAM171A2, prognostic association, histological heterogeneity, immune infiltration, bioinformatics, miRNA

## Abstract

**Background/Objectives:** Sarcomas represent a diverse group of mesenchymal malignancies characterized by unique histological, molecular, and immune features. FAM171A1 and FAM171A2, although related members of the FAM171 family, exhibit significant differences in their current biological characterization, with their relevance to sarcoma largely unexplored. This study conducted a comparative characterization of FAM171A1 and FAM171A2 using a comprehensive bioinformatic approach. **Methods:** This approach integrated data from TCGA/GTEx, GEPIA3, the Human Protein Atlas (HPA), TIMER/TIMER3, GeneMANIA, TargetScan, and ENCORI, alongside two independent Gene Expression Omnibus (GEO) cohorts with paired normal tissues. Systematic evaluations were performed on gene expression, histology-specific patterns, overall survival, immune infiltration, functional association networks, and predicted miRNA-related relationships. **Results:** Both genes demonstrated higher expression in the pooled SARC cohort compared to the normal-reference dataset, with significant expression heterogeneity observed across major histological subtypes. In independent cohorts with paired normal tissue, FAM171A2 upregulation was reproduced in a matched liposarcoma cohort (15 patients), whereas FAM171A1 upregulation was not. Higher expression of both genes correlated with poorer overall survival, and these associations remained significant in histology-stratified multivariable Cox models after adjusting for available clinical covariates. Exploratory subtype-specific analyses suggested an association with overall survival in undifferentiated pleomorphic sarcoma; however, this finding was based on a small subgroup with wide confidence intervals and requires independent validation. Immune analyses indicated histology-dependent and gene-specific patterns of immune-cell association, suggesting non-uniform immune relationships across sarcoma subtypes. GeneMANIA identified database-supported functional associations, while TargetScan and ENCORI identified candidate miRNA-related relationships; these findings are considered hypothesis-generating rather than mechanistic evidence. **Conclusions:** Overall, FAM171A1 and FAM171A2 display distinct, histology-dependent expression, survival, and immune-associated patterns in sarcoma. These findings represent prognostic associations rather than established prognostic biomarkers; they prioritize both genes for further investigation but do not establish functional, mechanistic, or therapeutic roles. Independent clinical validation and targeted experimental studies are necessary to ascertain their biological and potential prognostic relevance.

## 1. Introduction

Sarcomas are a heterogeneous group of malignant tumors arising from mesenchymal tissues, broadly classified into bone and soft tissue sarcomas, and comprising over 100 distinct subtypes [1]. These rare tumors occur across all age groups but are more common in adolescents and young adults, accounting for approximately 1% of adult malignancies and nearly 20% of pediatric malignancies [2,3]. Compared with many other cancers, sarcomas often exhibit a high degree of malignancy and a marked propensity for hematogenous metastasis to distant organs [4]. Standard treatment consists of surgery, chemotherapy, and radiotherapy; however, despite these multimodal treatment approaches and the emergence of targeted therapies and immunotherapy, clinical outcomes remain unsatisfactory [4,5]. Up to 50% of patients with sarcoma develop metastases within five years, and the 5-year survival rate for patients with distant metastasis remains approximately 16% [1]. These unfavorable clinical outcomes underscore the importance of elucidating the molecular mechanisms underlying sarcoma progression to facilitate the identification of novel biomarkers and potential therapeutic targets.

Among the diverse molecular classes investigated as cancer biomarkers and therapeutic targets, cell surface proteins have attracted considerable interest because of their essential roles in intercellular communication, signal transduction, and tumor progression [6]. The family with sequence similarity 171 (FAM171) comprises three type-I transmembrane cell surface proteins, namely family with sequence similarity 171 member A1 (FAM171A1), family with sequence similarity 171 member A2 (FAM171A2), and family with sequence similarity 171 member B (FAM171B) [7,8,9]. Members of this family have been implicated in several biological processes, including cell adhesion, cytoskeletal organization, intracellular trafficking, and signaling [7,9]. Among them, FAM171A1, also known as Astroprincin (APCN), is expressed in multiple tissues, including placental trophoblasts, skeletal and cardiac muscle, kidney, pancreas, and astrocytes [7]. Experimental studies have shown that FAM171A1 regulates cell morphology and promotes tumor cell invasion through modulation of actin cytoskeleton dynamics [7,10]. More recently, FAM171A1 has been identified as a hub gene and a potential prognostic biomarker and therapeutic target in breast cancer [10,11,12]. Furthermore, elevated FAM171A1 expression has been associated with poor prognosis in triple-negative breast cancer (TNBC) and colon cancer, as well as with increased tumor size and recurrence in oral tongue squamous cell carcinoma (OTSCC) [8,11,13]. In contrast, FAM171A2 remains considerably less characterized. It is predominantly expressed in the central nervous system, particularly in neurons, where it functions as a synapse-enriched cell-surface protein [14,15]. Recent studies have identified FAM171A2 as a novel neuronal receptor for pathological α-synuclein, mediating its endocytic uptake and facilitating cell-to-cell transmission, thereby highlighting its potential as a therapeutic target in Parkinson’s disease [14,16]. Although FAM171A2 has been implicated in neuronal signaling, vesicle trafficking, and extracellular matrix interactions, its biological role outside the nervous system, particularly in cancer, remains largely undefined. A recent bioinformatic study in gynecologic malignancies demonstrated that FAM171A2 exhibits a context-dependent expression pattern and is associated with neuronal and immune-related molecular networks [9]. Taken together, these findings suggest that FAM171A1 and FAM171A2 may have context-dependent biological functions, but their potential roles in sarcoma remain unknown.

The inclusion of FAM171A1 and FAM171A2 in the same analysis was not predicated on the assumption of functional equivalence due to their shared family membership. Instead, the significant differences in the existing biological evidence for these two related proteins justify their comparative investigation. FAM171A1 has been associated with cytoskeletal organization and tumor-related phenotypes, whereas FAM171A2 has been primarily characterized in neuronal biology and remains largely unexplored in cancer contexts. Evaluating these related yet biologically distinct family members in parallel offers an opportunity to ascertain whether they exhibit convergent or divergent patterns in expression, prognostic association, and immune association in sarcoma. Consequently, the present study was designed as a comparative, hypothesis-generating analysis rather than on the premise of equivalent functionality between FAM171A1 and FAM171A2. To our knowledge, FAM171A1 and FAM171A2 have not been previously investigated in sarcoma. The availability of large-scale public datasets facilitates the systematic exploration of understudied genes across molecular, clinical, immune, and regulatory dimensions. Therefore, this study aimed to comparatively characterize FAM171A1 and FAM171A2 in sarcoma and to determine whether these related family members exhibit convergent or divergent patterns of expression, prognostic association, immune infiltration, functional association networks, and predicted miRNA-related regulatory relationships using an integrated bioinformatic approach.

## 2. Materials and Methods

This study was conducted exclusively using multiple publicly available datasets and web-based bioinformatic platforms to investigate the expression patterns, prognostic associations, immune infiltration, functional interaction networks, and miRNA-related relationships of FAM171A1 and FAM171A2. Therefore, institutional ethics committee approval and informed consent were not required.

### 2.1. Gene Expression Analysis

Gene expression analysis of FAM171A1 and FAM171A2 was performed using the Gene Expression Profiling Interactive Analysis 3 (GEPIA3) online platform (https://gepia3.bioinfoliu.com/; accessed on 15 April 2026), which integrates transcriptomic data from The Cancer Genome Atlas (TCGA) and the Genotype-Tissue Expression (GTEx) projects [17]. The Boxplot module was used to compare mRNA expression levels between sarcoma (SARC) and normal tissues. Differential expression was evaluated using a threshold of |log2 fold change (FC)| > 1 and a q-value < 0.05. Gene expression levels were presented as log2(TPM + 1). To address the significant histological heterogeneity of sarcoma, subtype-specific expression analyses of FAM171A1 and FAM171A2 were conducted utilizing TCGA-SARC RNA-sequencing data and corresponding clinical annotations obtained via LinkedOmics. The gene-level RNA-sequencing data, generated using the Illumina HiSeq platform and processed through the RSEM pipeline, were analyzed as log2(RSEM + 1)-transformed expression values. Clinical annotations were aligned with RNA-sequencing samples using TCGA patient identifiers. Analyses were conducted at the gene level using the FAM171A1 and FAM171A2 expression estimates provided by the respective platforms; no individual transcript or alternatively spliced isoform was selected for the primary expression analyses. For GEPIA3, the SARC tumor dataset was compared with the normal-reference dataset available through the platform, which includes GTEx normal tissues. In the TCGA-SARC subtype analyses, only samples with matched RNA-sequencing and histological annotation data were retained. Duplicate or unmatched records, as well as samples that could not be assigned to the predefined histological categories, were excluded.

Histological diagnoses were standardized into six major sarcoma subtypes: dedifferentiated liposarcoma (DDLPS), leiomyosarcoma (LMS), myxofibrosarcoma (MFS), malignant peripheral nerve sheath tumor (MPNST), synovial sarcoma (SS), and undifferentiated pleomorphic sarcoma (UPS). Records annotated as pleomorphic malignant fibrous histiocytoma/undifferentiated pleomorphic sarcoma and related UPS terminology were consolidated into the UPS category. Monophasic, biphasic, and poorly differentiated synovial sarcoma annotations were combined into the SS category. Desmoid tumors were excluded from the primary subtype comparison. Following the matching of expression and clinical data and the application of these predefined histological categories, 257 tumor samples were included in the analysis (DDLPS, n = 58; LMS, n = 104; MFS, n = 25; MPNST, n = 9; SS, n = 10; and UPS, n = 51).

Differences in FAM171A1 and FAM171A2 expression across histological subtypes were evaluated using the Kruskal–Wallis test. Where applicable, pairwise post hoc comparisons were conducted with adjustments for multiple testing using the Benjamini–Hochberg false discovery rate (FDR) procedure. Adjusted *p*-values (q-values) < 0.05 were considered statistically significant. Expression distributions were visualized using boxplots with individual tumor samples superimposed.

### 2.2. Independent Validation Cohorts

Two independent Gene Expression Omnibus (GEO) datasets were used to evaluate tumor–normal expression differences outside the TCGA/GTEx framework. GSE198568 is an RNA-sequencing dataset comprising four paired soft-tissue sarcoma and adjacent normal tissue samples; expression values were expressed as counts per million (CPM), and paired differences were assessed using the exact two-sided Wilcoxon signed-rank test. GSE159659 (platform GPL23159, Affymetrix Clariom S Human Array; Affymetrix, Santa Clara, CA, USA) comprises 45 samples from 15 patients with retroperitoneal liposarcoma, including dedifferentiated (DDLPS) and well-differentiated (WDLPS) tumor components and normal adipose tissue obtained from the same tumor site of each patient [18]. RMA-normalized expression values were obtained from the series matrix, and transcript clusters were annotated using the GPL23159 platform file (FAM171A1: TC1000009884.hg.1; FAM171A2: TC1700010828.hg.1). Differential expression was assessed using a linear model including tissue type and patient as a blocking factor, followed by empirical Bayes variance moderation (limma). *p*-values were adjusted across all transcript clusters using the Benjamini–Hochberg procedure. Paired *t*-tests, Wilcoxon signed-rank tests, and the area under the receiver operating characteristic curve (AUC) were calculated as complementary analyses.

### 2.3. RNA Expression Analysis in Cancer Cell Lines

The RNA expression levels of FAM171A1 and FAM171A2 across human cancer cell lines were analyzed using the Human Protein Atlas (HPA) database (https://www.proteinatlas.org/; accessed on 15 April 2026). RNA expression data were obtained from the Cell Line section of the HPA and are presented as normalized transcripts per million (nTPM) [19]. RNA expression was first evaluated across different cancer cell line categories and subsequently analyzed in detail across 15 sarcoma-derived cell lines.

### 2.4. Survival Analysis

Survival analysis of FAM171A1 and FAM171A2 was performed using the Kaplan–Meier Plotter database (http://kmplot.com/analysis/; accessed on 15 April 2026). The pan-cancer RNA-sequencing dataset available on the platform was used to evaluate the associations between gene expression and overall survival (OS) and relapse-free survival (RFS) in patients with sarcoma [20]. For the initial exploratory Kaplan–Meier analyses, patients were divided into high- and low-expression groups using the automatically selected best-performing cutoff implemented by the Kaplan–Meier Plotter platform. Because data-driven cutoff optimization may increase the apparent strength of survival associations, these analyses were regarded as exploratory. Accordingly, the principal adjusted survival assessment was subsequently performed using multivariable Cox proportional hazards regression with gene expression modeled as a continuous variable, without dichotomization. Kaplan–Meier survival curves were generated by the platform, and differences between the two groups were evaluated using the log-rank test. Hazard ratios (HRs), 95% confidence intervals (CIs), and corresponding *p*-values were obtained directly from the platform. To assess whether the prognostic associations identified in the pooled SARC cohort were maintained within individual histological entities, exploratory subtype-specific overall survival analyses were conducted using matched TCGA-SARC RNA-sequencing and clinical data. The analyses focused on the three major histological subtypes with sufficient sample sizes and available survival data: LMS, DDLPS, and undifferentiated pleomorphic sarcoma (UPS). Smaller subtypes were excluded from separate analysis due to inadequate sample sizes and insufficient survival events for reliable estimation.

Within each histological subtype, patients were categorized into high- and low-expression groups for FAM171A1 and FAM171A2, using the subtype-specific median expression value as the threshold. Overall survival was defined based on the available TCGA follow-up time and vital-status information. Kaplan–Meier survival curves were generated, and differences between expression groups were assessed using the log-rank test. HRs and CIs comparing the high-expression group to the low-expression group were estimated using univariable Cox proportional hazards regression.

Given that six subtype-specific survival comparisons were conducted (three histological subtypes × two genes), the corresponding log-rank *p*-values were adjusted using the Benjamini–Hochberg FDR procedure. An FDR-adjusted q-value < 0.05 was deemed statistically significant. These subtype-specific analyses were considered exploratory due to the reduced sample sizes and numbers of survival events following histological stratification.

To evaluate whether the associations of FAM171A1 and FAM171A2 expression with overall survival persisted after adjusting for available clinicopathological variables and histological heterogeneity, multivariable Cox proportional hazards regression analyses were performed using matched TCGA-SARC RNA-sequencing and clinical data. Separate models were developed for FAM171A1 and FAM171A2. Gene expression was modeled continuously as log_2_(RSEM + 1), thereby minimizing information loss and potential bias associated with data-driven dichotomization. The primary models were adjusted for age (continuous) and sex and stratified by histological subtype (LMS, DDLPS, UPS, MFS, SS, and MPNST), allowing the baseline hazard function to vary across histological subtypes without estimating a common hazard ratio for histology. Complete-case analysis was employed, resulting in 252 patients and 95 death events. Sensitivity models additionally included radiotherapy and residual tumor status while retaining histological stratification; these analyses included 246 patients and 94 death events. HRs with CIs were reported for a one-unit increase in log_2_(RSEM + 1) gene expression. Statistical tests were two-sided, and *p* < 0.05 was considered statistically significant. Proportional-hazards assumptions were evaluated using Schoenfeld residual-based diagnostics.

### 2.5. Immune Infiltration Analysis

Immune infiltration analysis of FAM171A1 and FAM171A2 was performed using the Tumor IMmune Estimation Resource (TIMER) database (https://cistrome.shinyapps.io/timer/; accessed on 15 April 2026). The Gene module was used to evaluate the correlations between gene expression and the infiltration of B cells, CD8^+^ T cells, CD4^+^ T cells, macrophages, neutrophils, and dendritic cells in sarcoma tissues. Correlations were assessed using purity-corrected partial Spearman’s correlation analysis [21]. To further investigate the association between gene expression and regulatory T-cell (Treg) infiltration, the TIMER3 platform (https://compbio.cn/timer3/; accessed on 3 July 2026) was used. The Gene module was employed to estimate Treg infiltration using multiple immune deconvolution algorithms, including CIBERSORT, CIBERSORT-ABS, ConsensusTME, quanTIseq, and xCell, with the Purity Adjustment option enabled when applicable. Correlations between gene expression and Treg infiltration were assessed using Spearman’s correlation analysis [22]. For all analyses, a *p* value < 0.05 was considered statistically significant.

To determine whether the associations between FAM171A1/FAM171A2 expression and the immune microenvironment vary by sarcoma histology, a subtype-specific immune analysis was conducted utilizing sample-level immune infiltration scores from the TCGA-SARC dataset. These immune scores were aligned with RNA-sequencing expression and histological annotation data through TCGA sample identifiers. To mitigate the risk of unstable correlation estimates due to small subgroup sizes, the analysis was confined to histological groups with a minimum of 20 matched samples. Consequently, LMS (n = 82) and DDLPS (n = 46) were included in the primary subtype-specific immune analysis. UPS was excluded from inferential analysis because immune-score data were available for only six matched cases, despite a larger number of UPS cases in the expression and survival datasets. Within each eligible histological subtype, the associations between FAM171A1 or FAM171A2 expression and 22 immune-cell infiltration scores were evaluated using Spearman rank correlation analysis. To address multiple comparisons, *p*-values were adjusted separately within each gene–histology analysis family using the Benjamini–Hochberg FDR procedure. FDR-adjusted q-values < 0.05 were deemed statistically significant. Correlation coefficients were depicted using a heatmap.

### 2.6. Gene Functional Network Analysis

Gene functional networks analysis of FAM171A1 and FAM171A2 was performed using the GeneMANIA database (https://genemania.org/; accessed on 20 April 2026). GeneMANIA integrates multiple sources of biological evidence to identify functionally associated genes and predict gene functions, including co-expression, physical interactions, genetic interactions, co-localization, pathway associations, and shared protein domains [23]. GeneMANIA served as an exploratory tool for network analysis to identify functional associations supported by databases. The associations, including co-expression, physical interactions, genetic interactions, and shared protein domains, were interpreted at the network level rather than as direct evidence of functional or mechanistic regulation in sarcoma. Separate queries for FAM171A1 and FAM171A2 were conducted in GeneMANIA, using Homo sapiens as the reference organism. The network was generated via the GeneMANIA web interface, utilizing interaction evidence categories such as co-expression, physical interaction, genetic interaction, co-localization, pathway, and shared protein-domain data. Default settings for network weighting and gene selection in GeneMANIA were maintained unless specified otherwise. The interaction type and corresponding network weight provided by GeneMANIA were documented for each associated gene, forming the basis for Table 1 and Table 2. No minimum threshold for interaction weight was applied post-network generation. The results from GeneMANIA were interpreted strictly as database-supported functional associations, not as evidence of direct molecular or mechanistic interactions in sarcoma.

### 2.7. microRNA Target Prediction

miRNA target prediction for FAM171A1 and FAM171A2 was performed using TargetScanHuman Release 8.0 (https://www.targetscan.org/vert_80/; accessed on 20 April 2026). TargetScan predicts miRNA–mRNA interactions by identifying conserved 8mer, 7mer, and 6mer binding sites complementary to the miRNA seed region within the 3′ untranslated regions (3′ UTRs) of target transcripts [24]. FAM171A1 and FAM171A2 were individually analyzed using TargetScanHuman Release 8.0, employing human gene symbols. The analysis was confined to miRNAs with conserved canonical seed-matched sites as identified by TargetScan within annotated 3′UTRs. The site types considered included canonical 8mer, 7mer-m8, 7mer-A1, and 6mer, as reported by the platform. It is important to note that no experimentally validated interactions were deduced solely from TargetScan predictions. In instances where multiple transcript/3′UTR annotations were present, the transcript representation provided by TargetScan for the respective gene-level query was utilized; no post hoc transcript selection was made based on the predicted miRNA outcomes.

### 2.8. Assessment of miRNA–FAM171A1/FAM171A2 Expression Correlations

miRNA–target co-expression analysis of FAM171A1 and FAM171A2 was performed using the Encyclopedia of RNA Interactomes (ENCORI) database (https://rnasysu.com/encori/panCancer.php; accessed on 20 April 2026). ENCORI is a pan-cancer platform that integrates RNA-sequencing and miRNA-sequencing data from TCGA and provides tools for analyzing miRNA–target co-expression networks [25]. ENCORI served as a supplementary tool for expression-correlation analysis of miRNAs identified in the prior TargetScan prediction phase. Consequently, miRNAs were not selected through an independent data-driven screening of the SARC cohort; rather, only the predefined candidates predicted by TargetScan were assessed in relation to their respective FAM171A1 or FAM171A2 target genes. The TCGA-SARC cohort, accessible via the ENCORI pan-cancer module, was chosen for analysis, which utilized matched mRNA-sequencing and miRNA-sequencing data. Correlation coefficients and their corresponding *p*-values were directly obtained from the platform. miRNA expression values were expressed as log_2_(RPM + 0.01). No criterion regarding the direction of correlation was applied; thus, both positive and negative correlations were included and reported. ENCORI correlations were interpreted solely as associations at the expression level and not as confirmation of direct miRNA-mediated regulation.

## 3. Results

### 3.1. Gene Expression Levels of FAM171A1 and FAM171A2 in Sarcoma Tissues

**Figure 1:**
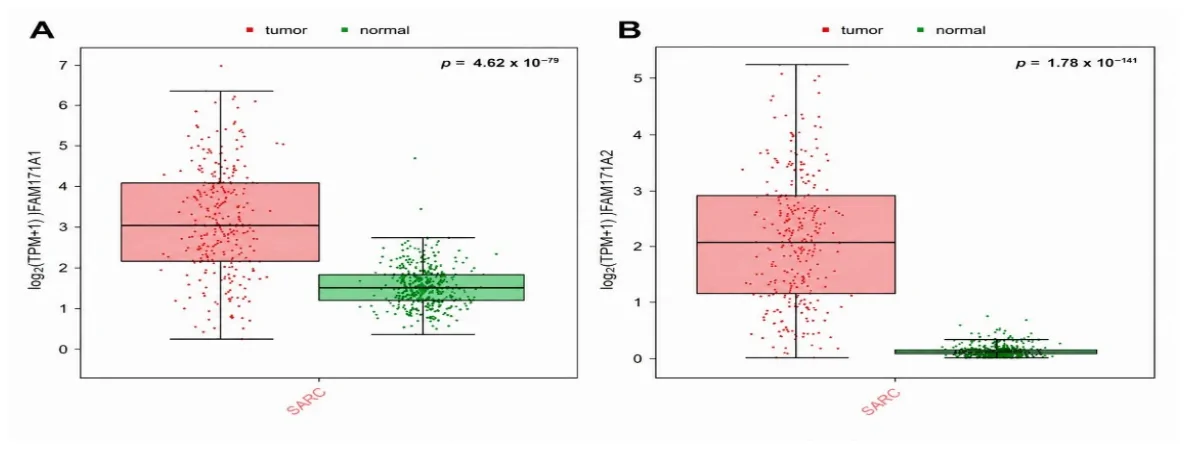
Differential expression of FAM171A1 and FAM171A2 in sarcoma (SARC) based on GEPIA3 analysis. (**A**) FAM171A1 and (**B**) FAM171A2 expression in SARC (tumor, red; n = 262) and normal tissues (green; n = 398). Expression levels are presented as log_2_(TPM + 1).

Gene expression analysis revealed that FAM171A1 was significantly upregulated in SARC tissues (n = 262) compared with normal tissues (n = 398), with median expression values of 3.00 and 1.49, respectively (*p* = 4.62 × 10^−79^; Figure 1A). Similarly, FAM171A2 was significantly upregulated in SARC tissues compared with normal tissues, with median expression values of 2.06 and 0.11, respectively (*p* = 1.78 × 10^−141^; Figure 1B).

**Figure 2:**
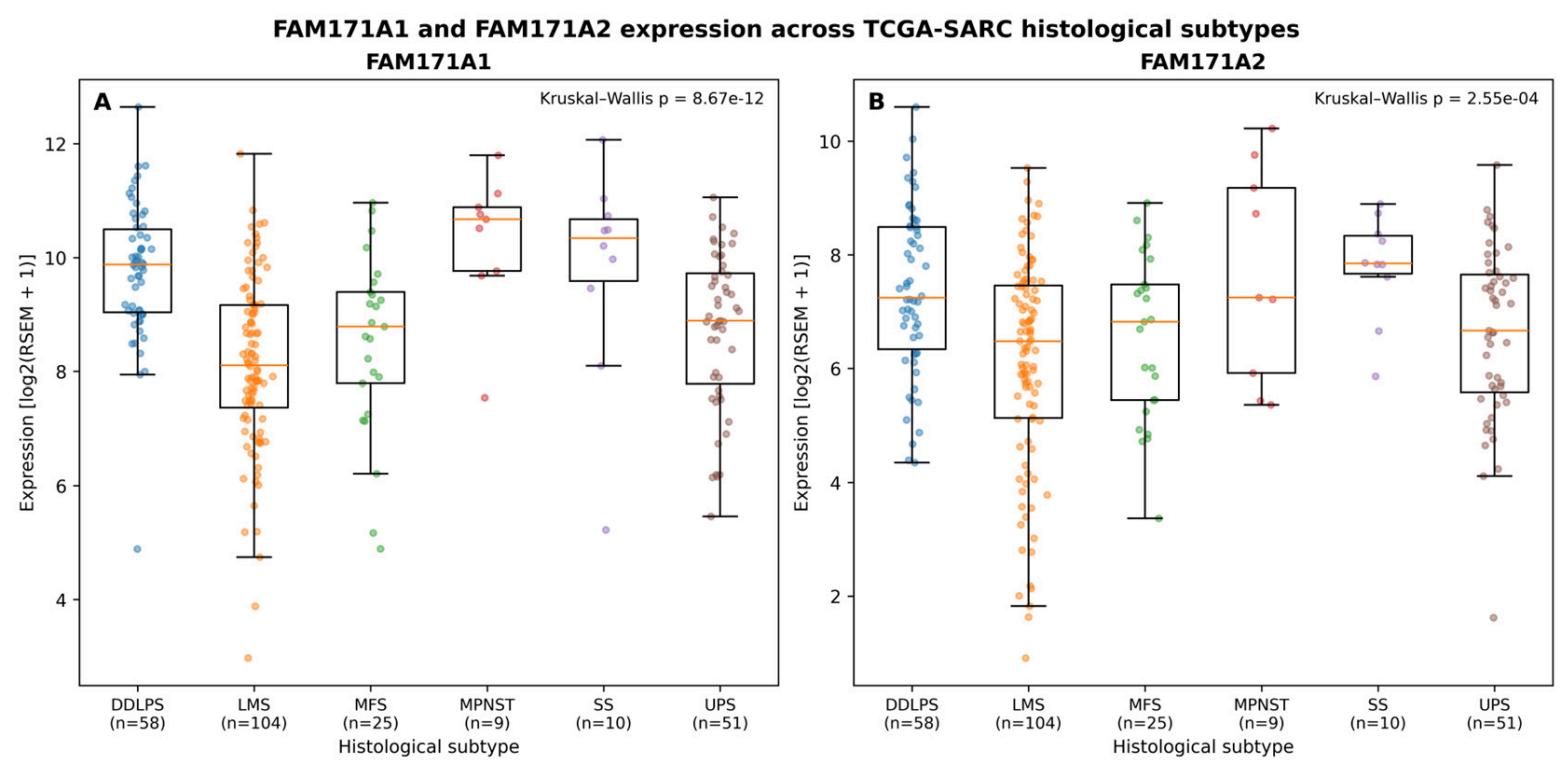
Histological subtype-specific expression of FAM171A1 and FAM171A2 in the TCGA-SARC cohort. Boxplots show the distribution of log2(RSEM + 1)-transformed expression levels of (**A**) FAM171A1 and (**B**) FAM171A2 across dedifferentiated liposarcoma (DDLPS; n = 58), leiomyosarcoma (LMS; n = 104), myxofibrosarcoma (MFS; n = 25), malignant peripheral nerve sheath tumor (MPNST; n = 9), synovial sarcoma (SS; n = 10), and undifferentiated pleomorphic sarcoma (UPS; n = 51). Individual points represent individual tumor samples. Overall differences among histological subtypes were assessed using the Kruskal–Wallis test. Significant subtype-associated heterogeneity was observed for both FAM171A1 (*p* = 8.67 × 10^−12^) and FAM171A2 (*p* = 2.55 × 10^−4^).

In order to address the histological heterogeneity of sarcoma, the expression of FAM171A1 and FAM171A2 was further assessed across six major histological subtypes within the TCGA-SARC cohort: dedifferentiated liposarcoma (DDLPS, n = 58), leiomyosarcoma (LMS, n = 104), myxofibrosarcoma (MFS, n = 25), malignant peripheral nerve sheath tumor (MPNST, n = 9), synovial sarcoma (SS, n = 10), and undifferentiated pleomorphic sarcoma (UPS, n = 51). The expression of FAM171A1 varied significantly among these histological subtypes (Kruskal–Wallis H = 60.71, *p* = 8.67 × 10^−12^). Notably, relatively high median expression levels were detected in MPNST, SS, and DDLPS, whereas LMS exhibited the lowest median expression. Similarly, FAM171A2 expression demonstrated significant variation across histological subtypes (H = 23.64, *p* = 2.55 × 10^−4^), with relatively elevated expression in SS and DDLPS, and reduced expression in LMS (Figure 2). Because the MPNST (n = 9) and SS (n = 10) groups were substantially smaller than the major histological groups, observations involving these subtypes were considered exploratory and are not interpreted as definitive subtype-specific findings.

**Figure 3:**
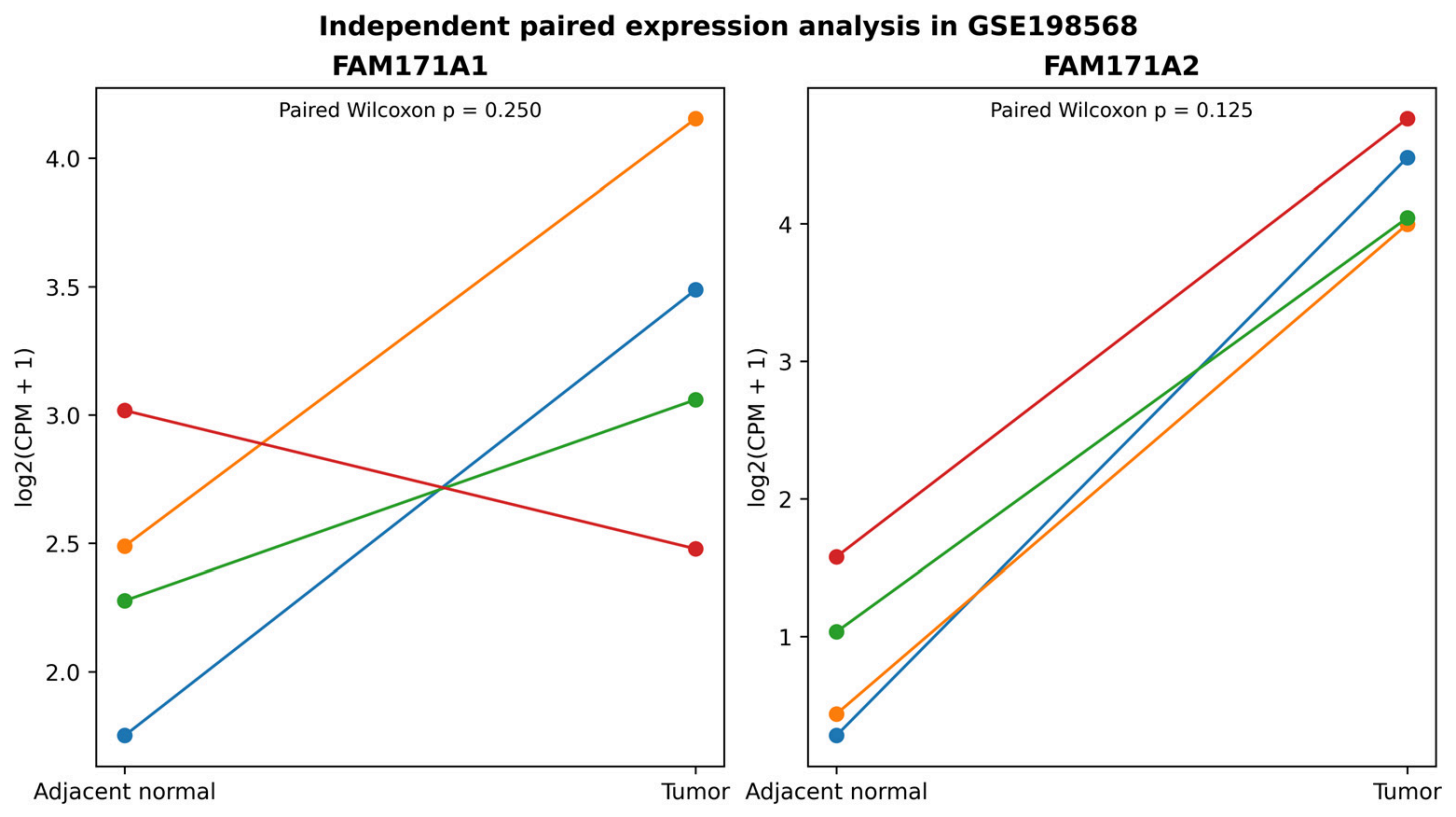
Independent paired expression analysis of FAM171A1 and FAM171A2 in the GSE198568 soft-tissue sarcoma cohort. Paired expression levels of FAM171A1 and FAM171A2 were compared between adjacent normal and tumor tissues from four patients. Each colored line connects the matched adjacent normal and tumor sample from the same patient, and points indicate individual sample expression values. Expression levels are presented as log_2_(CPM + 1). For FAM171A1, tumor expression was higher in three of four matched pairs, whereas one pair showed lower expression in the tumor sample. For FAM171A2, expression was higher in all four tumor samples compared with their corresponding adjacent normal tissues. Paired differences were evaluated using the exact two-sided Wilcoxon signed-rank test; neither comparison reached statistical significance (FAM171A1, *p* = 0.250; FAM171A2, *p* = 0.125). Given the limited number of matched samples, these findings should be interpreted as directional supportive evidence rather than statistically conclusive external validation.

To independently assess expression patterns beyond the TCGA/GTEx comparison, the expression of FAM171A1 and FAM171A2 was analyzed using the GSE198568 RNA-sequencing dataset, which includes four paired samples of soft-tissue sarcoma and adjacent normal tissue. In three out of the four matched pairs, FAM171A1 expression was elevated in tumor tissues, with median expression levels rising from 4.23 CPM in adjacent normal tissues to 8.78 CPM in tumors, resulting in a median tumor-to-normal ratio of 2.77-fold. FAM171A2 exhibited a more consistent pattern, with increased expression in all four tumor samples compared to their matched normal tissues (median, 18.39 vs. 0.70 CPM; median tumor-to-normal ratio, 28.47-fold). However, due to the limited number of paired samples, neither comparison achieved statistical significance according to the exact paired Wilcoxon signed-rank test (FAM171A1, *p* = 0.250; FAM171A2, *p* = 0.125). Consequently, these findings offer directional, albeit not statistically definitive, support for the expression patterns identified in the initial analysis (Figure 3).

**Figure 4:**
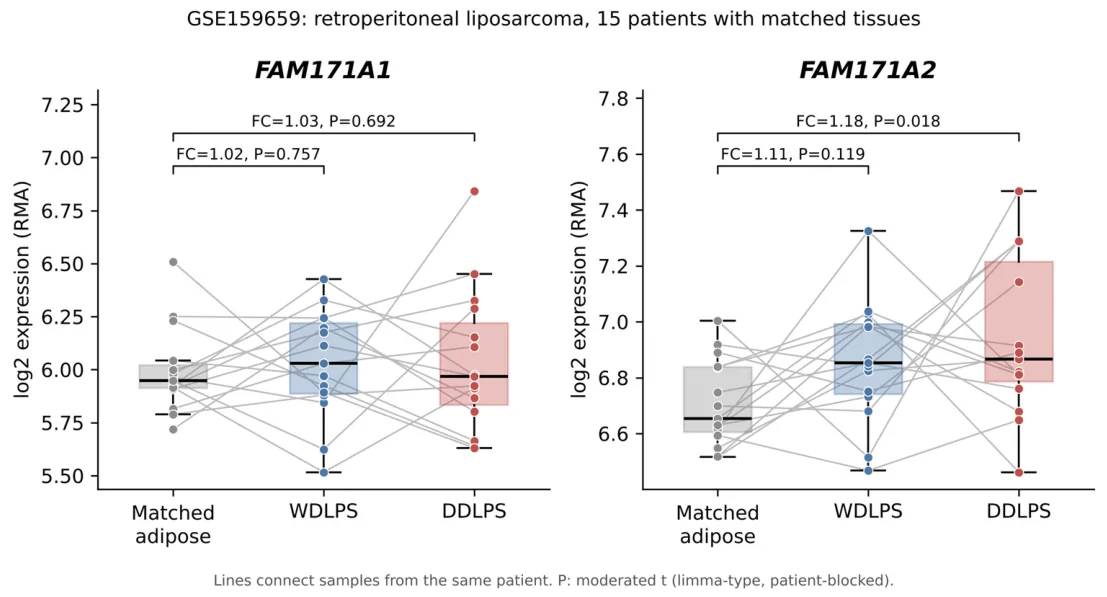
FAM171A1 and FAM171A2 expression in the independent GSE159659 cohort (n = 15 patients). Matched adipose tissue, well-differentiated (WDLPS) and dedifferentiated liposarcoma (DDLPS) samples from each patient are connected by lines. Fold changes and *p* values were derived from a patient-blocked linear model with empirical Bayes moderation.

To further evaluate these findings in a larger cohort with anatomically matched normal tissue, we analyzed GSE159659, which includes DDLPS, WDLPS, and normal adipose tissue samples obtained from the same tumor site in 15 patients with retroperitoneal liposarcoma [18]. In this matched cohort, FAM171A2 expression was higher in tumor tissue than in patient-matched adipose tissue, consistent with the direction observed in our primary analysis. The increase was most evident in DDLPS (fold change 1.18, moderated *p* = 0.018; paired *t*-test *p* = 0.006; higher in 13 of 15 patients; AUC = 0.74), whereas the difference in WDLPS did not reach statistical significance (fold change 1.11, *p* = 0.119). The association for FAM171A2 did not remain significant after genome-wide multiple-testing correction (FDR = 0.069). In contrast, FAM171A1 expression did not differ between tumor and matched adipose tissue (DDLPS: fold change 1.03, *p* = 0.69; WDLPS: fold change 1.02, *p* = 0.76; AUC ≈ 0.50) (Figure 4).

### 3.2. RNA Expression Levels of FAM171A1 and FAM171A2 in Sarcoma Cell Lines

**Figure 5:**
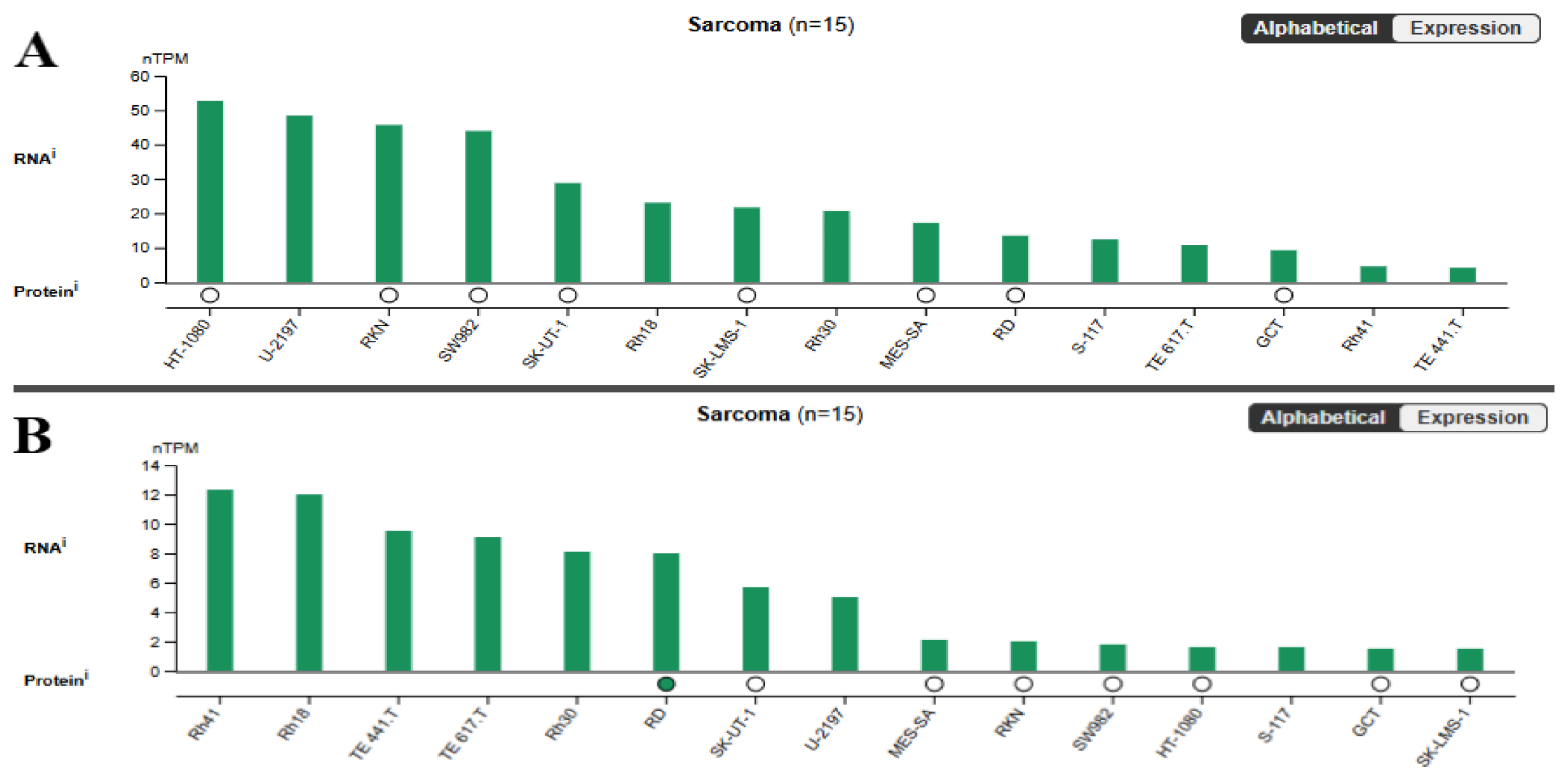
RNA expression profiles of FAM171A1 and FAM171A2 across human cancer cell lines based on the Human Protein Atlas database. (**A**) RNA expression of FAM171A1 in 15 sarcoma cell lines. (**B**) RNA expression of FAM171A2 in 15 sarcoma cell lines. RNA expression levels are presented as normalized transcripts per million (nTPM).

RNA expression analysis using the HPA revealed that FAM171A1 and FAM171A2 were expressed across multiple human cancer cell line categories, including sarcoma cell lines. Detailed analysis of the 15 sarcoma cell lines revealed that FAM171A1 exhibited the highest expression in HT-1080 (52.7 nTPM), whereas FAM171A2 showed the highest expression in RH41 (12.3 nTPM) (Figure 5A,B).

### 3.3. Prognostic Associations of FAM171A1 and FAM171A2 in Sarcoma

**Figure 6:**
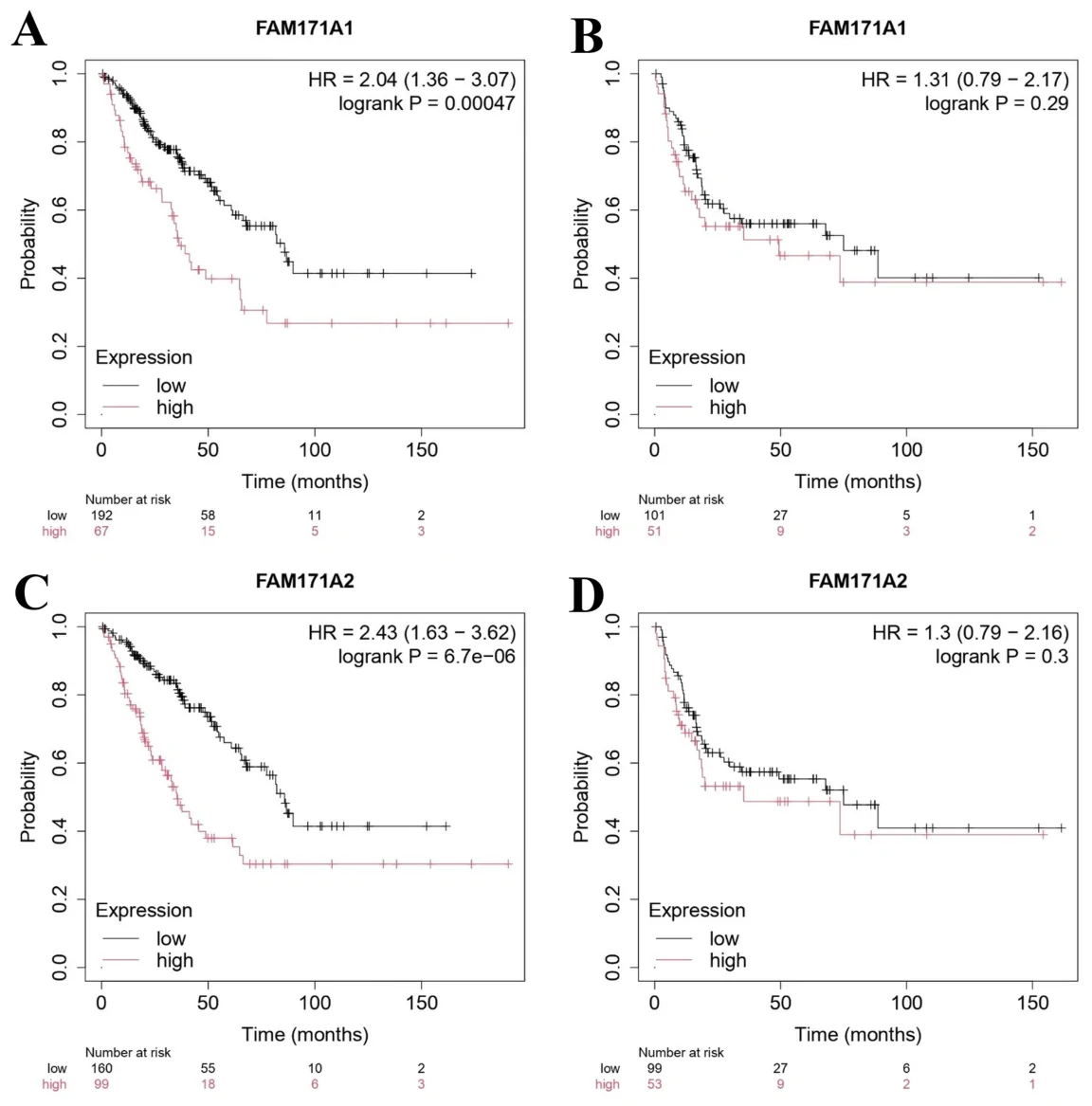
Kaplan–Meier survival analysis of FAM171A1 and FAM171A2 expression in patients with sarcoma using the Kaplan–Meier Plotter database. (**A**) Overall survival (OS) analysis of FAM171A1. (**B**) Relapse-free survival (RFS) analysis of FAM171A1. (**C**) OS analysis of FAM171A2. (**D**) RFS analysis of FAM171A2. The red and black curves represent the high- and low-expression groups, respectively. HR, hazard ratio; CI, confidence interval; log-rank *p*, *p*-value obtained from the log-rank test. Patients were dichotomized using the best-performing cutoff automatically selected by the platform; because this approach may overestimate prognostic associations, these analyses are considered exploratory.

In exploratory Kaplan–Meier analyses based on the automatically selected best-performing cutoff, high FAM171A1 expression was significantly associated with poorer OS in patients with sarcoma (n = 259) (HR) = 2.04, 95% CI = 1.36–3.07, log-rank *p* = 0.00047; Figure 6A). The median survival time was 85.83 months in the low-expression group and 36.27 months in the high-expression group. However, FAM171A1 expression was not significantly associated with RFS (HR = 1.31, 95% CI = 0.79–2.17, log-rank *p* = 0.29; Figure 6B). Similarly, high FAM171A2 expression was significantly associated with poorer OS (HR = 2.43, 95% CI = 1.63–3.62, log-rank *p* = 6.7 × 10^−6^; Figure 6C). The median survival time was 85.83 months in the low-expression group and 35.37 months in the high-expression group. In contrast, FAM171A2 expression was not significantly associated with RFS (HR = 1.30, 95%, CI = 0.79–2.16, log-rank *p* = 0.30; Figure 6D). Because data-driven cutoff selection may overestimate the strength of prognostic associations, these results were considered exploratory, and the continuous-variable multivariable Cox analyses described below were regarded as the main survival analysis.

Subsequent exploratory survival analyses specific to subtypes were conducted for LMS, DDLPS, and UPS, which had adequate sample sizes and available survival data. After excluding cases lacking survival information, the analyses comprised 102 patients with LMS, 57 with DDLPS, and 51 with UPS.

In the LMS cohort, neither FAM171A1 (HR = 1.22, 95% CI = 0.65–2.27; log-rank *p* = 0.534) nor FAM171A2 (HR = 1.75, 95% CI = 0.93–3.29; log-rank *p* = 0.077) demonstrated a significant association with overall survival. Similarly, in DDLPS, no statistically significant association was found for FAM171A1 (HR = 1.98, 95% CI = 0.87–4.53; log-rank *p* = 0.099) or FAM171A2 (HR = 1.35, 95% CI = 0.60–3.03; log-rank *p* = 0.467).

**Figure 7:**
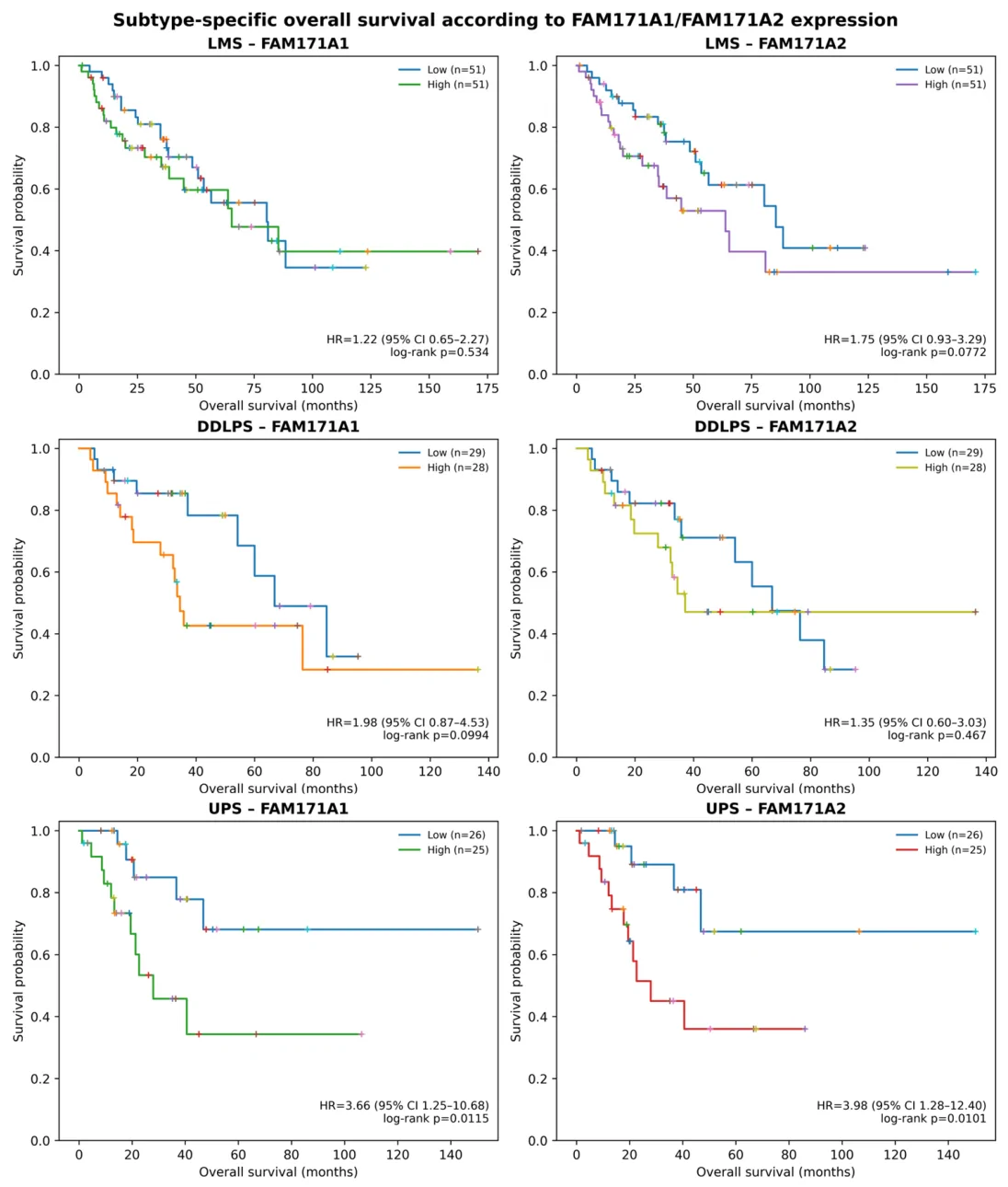
Histological subtype-specific overall survival according to FAM171A1 and FAM171A2 expression in the TCGA-SARC cohort. Kaplan–Meier curves show overall survival according to high versus low expression of FAM171A1 and FAM171A2 in leiomyosarcoma (LMS), dedifferentiated liposarcoma (DDLPS), and undifferentiated pleomorphic sarcoma (UPS). Patients were dichotomized using the subtype-specific median gene-expression value. No significant survival associations were observed for either gene in LMS or DDLPS. In UPS, high expression of FAM171A1 (HR = 3.66, 95% CI = 1.25–10.68; log-rank *p* = 0.0115) and FAM171A2 (HR = 3.98, 95% CI = 1.28–12.40; log-rank *p* = 0.0101) was significantly associated with poorer overall survival. Both UPS associations remained significant after Benjamini–Hochberg FDR correction (q = 0.0346). Tick marks indicate censored observations.

Conversely, in UPS, elevated expression levels of both genes were significantly correlated with poorer overall survival. High expression of FAM171A1 was linked to an approximately 3.7-fold increased hazard of death compared to low expression (HR = 3.66, 95% CI = 1.25–10.68; log-rank *p* = 0.0115), while high expression of FAM171A2 was associated with an approximately 4.0-fold increased hazard (HR = 3.98, 95% CI = 1.28–12.40; log-rank *p* = 0.0101). These associations remained statistically significant following Benjamini–Hochberg correction for the six subtype-specific survival comparisons (FDR q = 0.0346 for each gene) (Figure 7). However, the confidence intervals were wide, reflecting the limited number of patients and events in this subgroup; these findings should therefore be regarded as preliminary and require independent validation.

**Figure 8:**
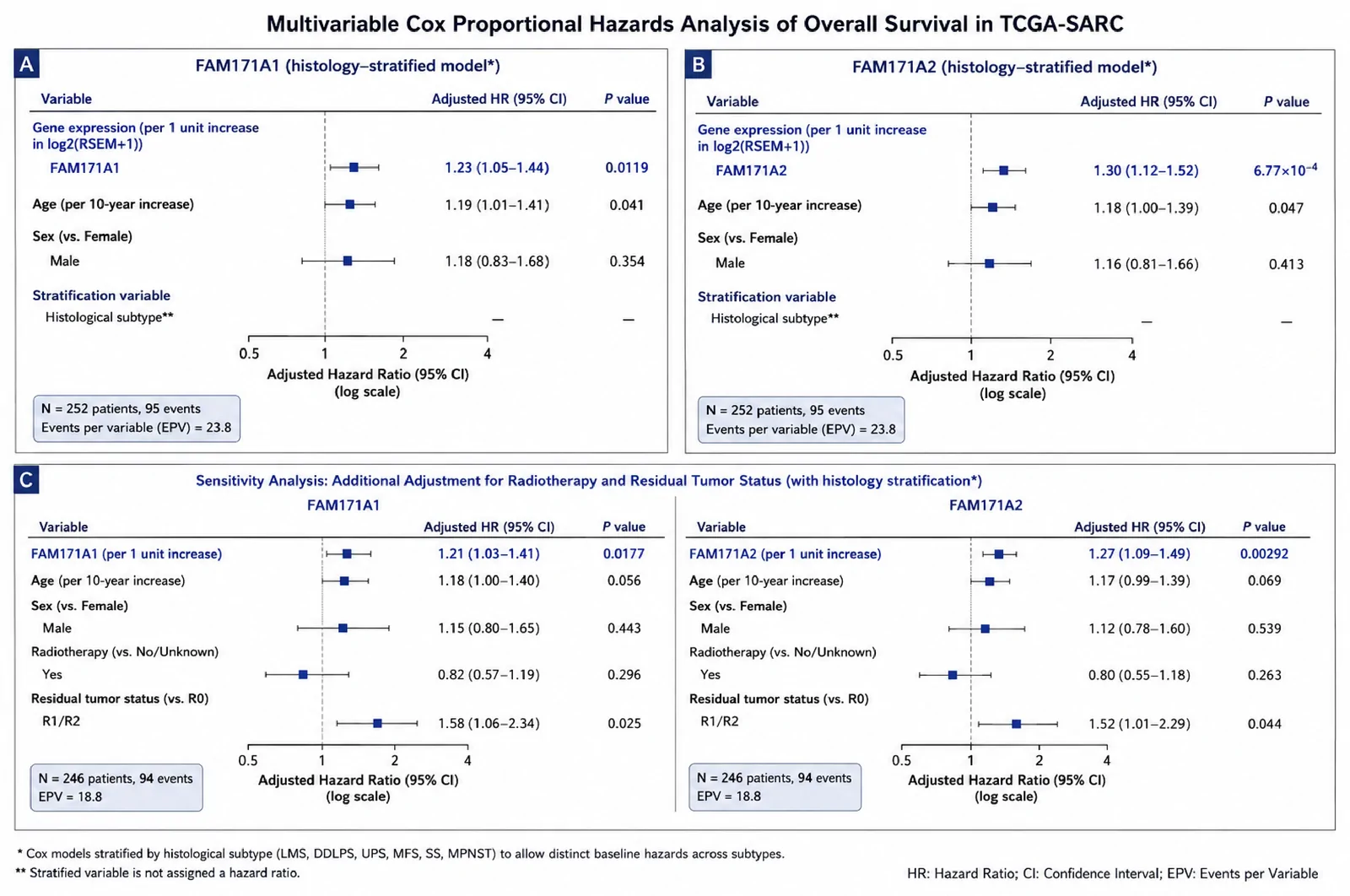
Multivariable Cox proportional hazards analyses of overall survival according to FAM171A1 and FAM171A2 expression in the TCGA-SARC cohort. (**A**) Histology-stratified multivariable Cox regression analysis for FAM171A1. Higher FAM171A1 expression, modeled as a continuous variable per 1-unit increase in log_2_(RSEM + 1), was significantly associated with an increased hazard of death after adjustment for age and sex (adjusted HR = 1.23, 95% CI = 1.05–1.44; *p* = 0.0119). (**B**) Corresponding analysis for FAM171A2 demonstrated a significant association between higher expression and increased mortality risk (adjusted HR = 1.30, 95% CI = 1.12–1.52; *p* = 6.77 × 10^−4^). Both primary models included 252 patients and 95 death events and were stratified by histological subtype (LMS, DDLPS, UPS, MFS, SS, and MPNST), allowing the baseline hazard to vary across histological subtypes. (**C**) Sensitivity analyses additionally adjusted for radiotherapy and residual tumor status while retaining histological subtype stratification. The associations remained significant for both FAM171A1 (adjusted HR = 1.21, 95% CI = 1.03–1.41; *p* = 0.0177) and FAM171A2 (adjusted HR = 1.27, 95% CI = 1.09–1.49; *p* = 0.00292). Sensitivity models included 246 patients and 94 death events. Squares represent adjusted hazard ratios and horizontal lines indicate 95% confidence intervals; the vertical reference line at HR = 1 indicates no association. Histological subtype was used as a stratification variable and therefore was not assigned an individual hazard ratio. HR, hazard ratio; CI, confidence interval; EPV, events per variable; LMS, leiomyosarcoma; DDLPS, dedifferentiated liposarcoma; UPS, undifferentiated pleomorphic sarcoma; MFS, myxofibrosarcoma; SS, synovial sarcoma; MPNST, malignant peripheral nerve sheath tumor.

To determine whether the associations between FAM171A1/FAM171A2 expression and overall survival persisted after accounting for histological heterogeneity and available clinical covariates, histology-stratified multivariable Cox proportional hazards models, which constituted the main survival analysis, were performed with gene expression modeled as a continuous variable. In the primary models, which included 252 patients and 95 death events and were adjusted for age and sex while allowing the baseline hazard to vary across histological subtypes, higher FAM171A1 expression was significantly associated with an increased hazard of death (adjusted HR = 1.23, 95% CI = 1.05–1.44; *p* = 0.0119). Similarly, higher FAM171A2 expression was significantly associated with poorer overall survival (adjusted HR = 1.30, 95% CI = 1.12–1.52; *p* = 6.77 × 10^−4^). In sensitivity analyses additionally adjusted for radiotherapy and residual tumor status, the associations remained significant for FAM171A1 (adjusted HR = 1.21, 95% CI = 1.03–1.41; *p* = 0.0177) and FAM171A2 (adjusted HR = 1.27, 95% CI = 1.09–1.49; *p* = 0.00292). These findings indicate that the observed survival associations were not solely explained by histological composition or the available clinical covariates; nevertheless, they should be interpreted as adjusted prognostic associations rather than evidence of established clinical biomarkers (Figure 8).

### 3.4. Correlation Between FAM171A1 and FAM171A2 Expression and Immune Infiltration

**Figure 9:**
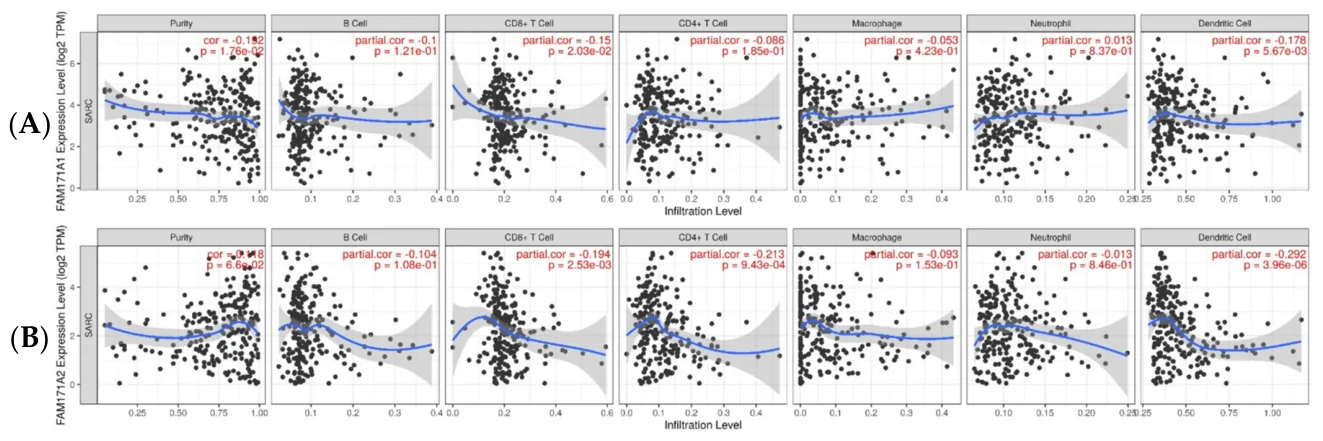
Correlations between FAM171A1 and FAM171A2 expression and immune cell infiltration in sarcoma based on the TIMER database. (**A**) Correlations between FAM171A1 expression and the infiltration of six major immune cell types estimated by TIMER. (**B**) Correlations between FAM171A2 expression and the infiltration of six major immune cell types estimated by TIMER.

TIMER analysis revealed that FAM171A1 expression in sarcoma was negatively correlated with tumor purity (partial correlation = −0.152, *p* = 1.76 × 10^−2^). Significant negative correlations were also observed with CD8^+^ T cells (partial correlation = −0.150, *p* = 2.03 × 10^−2^) and dendritic cells (partial correlation = −0.178, *p* = 5.67 × 10^−3^). No significant correlations were detected with B cells, CD4^+^ T cells, macrophages, or neutrophils (*p* > 0.05) (Figure 9A). Treg infiltration analysis using multiple immune deconvolution algorithms demonstrated that FAM171A1 expression was significantly negatively correlated with Treg infiltration estimated by CIBERSORT (rho = −0.172, *p* = 7.14 × 10^−3^), CIBERSORT-ABS (rho = −0.208, *p* = 1.08 × 10^−3^), and ConsensusTME (rho = −0.201, *p* = 1.60 × 10^−3^). No significant associations were observed using quanTIseq (rho = −0.095, *p* = 0.142) or xCell (rho = 0.092, *p* = 0.151). Similarly, FAM171A2 expression was negatively correlated with CD8^+^ T cells (partial correlation = −0.194, *p* = 2.53 × 10^−3^), CD4^+^ T cells (partial correlation = −0.213, *p* = 9.43 × 10^−4^), and dendritic cells (partial correlation = −0.292, *p* = 3.96 × 10^−6^). No significant correlations were observed with tumor purity, B cells, macrophages, or neutrophils (*p* > 0.05) (Figure 9B). Treg infiltration analysis demonstrated that the association between FAM171A2 expression and Treg infiltration varied according to the immune deconvolution algorithms. No significant associations were observed using CIBERSORT (rho = −0.083, *p* = 0.198) or xCell (rho = 0.014, *p* = 0.827), whereas significant negative correlations were identified using CIBERSORT-ABS (rho = −0.175, *p* = 6.28 × 10^−3^), ConsensusTME (rho = −0.306, *p* = 1.20 × 10^−6^), and quanTIseq (rho = −0.150, *p* = 1.97 × 10^−2^).

**Figure 10:**
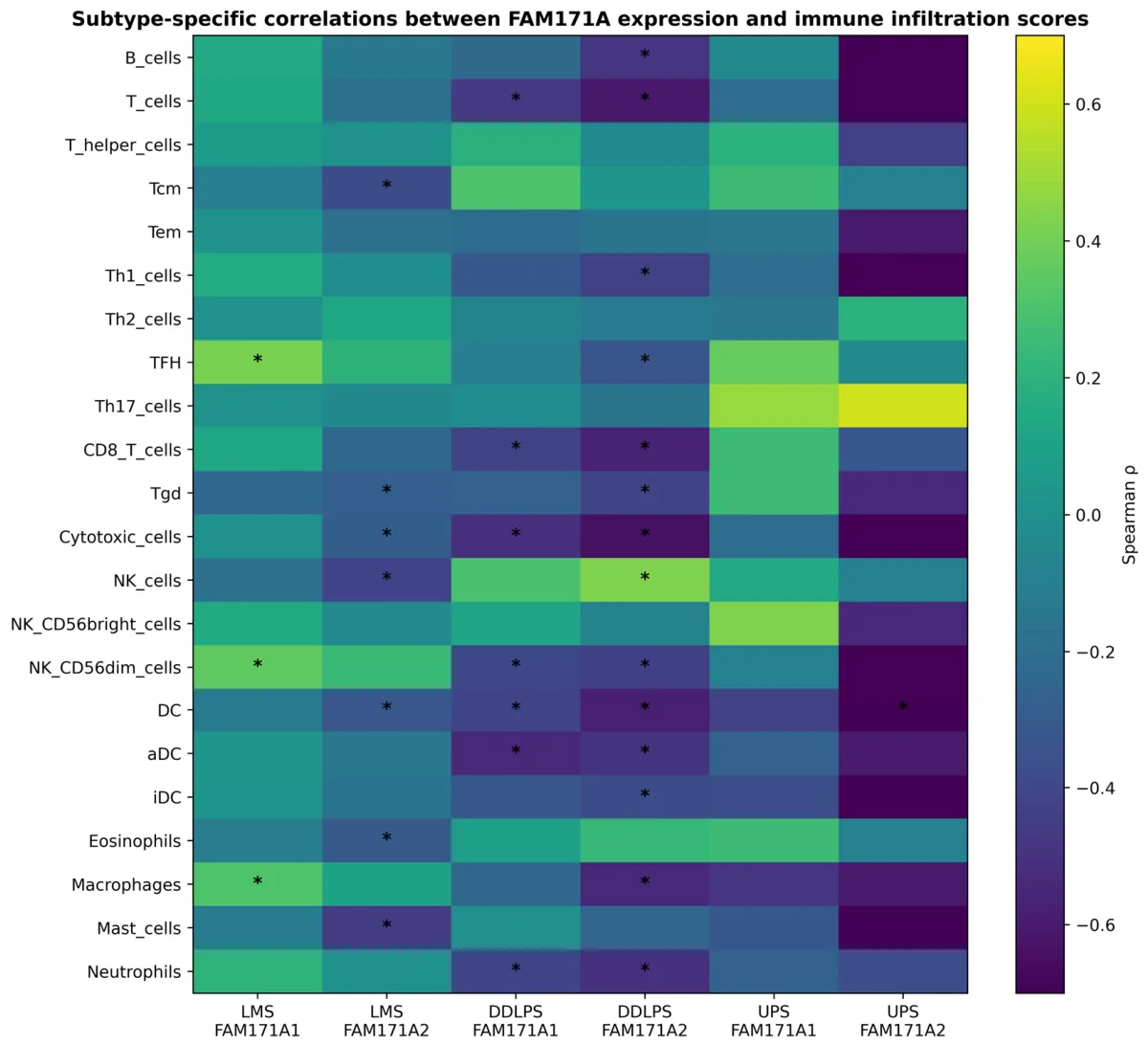
Histological subtype-specific associations between FAM171A1/FAM171A2 expression and immune infiltration in TCGA-SARC. Heatmap showing Spearman correlation coefficients (ρ) between FAM171A1 or FAM171A2 expression and immune-cell infiltration scores in histological subtypes with sufficient matched immune data. LMS and DDLPS included 82 and 46 matched tumors, respectively. Asterisks indicate correlations remaining significant after Benjamini–Hochberg FDR correction (q < 0.05). UPS was not included in inferential interpretation because only six tumors had matched immune-score data.

Subtype-specific immune analyses identified distinct associations between the expression of FAM171A1/FAM171A2 and immune infiltration in LMS and DDLPS. In DDLPS (n = 46), FAM171A1 expression exhibited an inverse correlation with several immune-cell scores, including activated dendritic cells (aDC; ρ = −0.538, FDR q = 0.0025), cytotoxic cells (ρ = −0.505, q = 0.0038), T cells (ρ = −0.467, q = 0.0079), CD8 T cells (ρ = −0.414, q = 0.0166), dendritic cells (ρ = −0.412, q = 0.0166), and neutrophils (ρ = −0.411, q = 0.0166). FAM171A2 demonstrated an even broader inverse immune association in DDLPS, with the strongest correlations observed for cytotoxic cells (ρ = −0.638, q = 4.1 × 10^−5^), T cells (ρ = −0.605, q = 9.2 × 10^−5^), dendritic cells (ρ = −0.574, q = 2.1 × 10^−4^), CD8 T cells (ρ = −0.568, q = 2.1 × 10^−4^), and macrophages (ρ = −0.537, q = 5.3 × 10^−4^). A different pattern emerged in LMS (n = 82), where FAM171A1 expression was positively correlated with TFH cells (ρ = 0.420, q = 0.0019), NK CD56dim cells (ρ = 0.351, q = 0.0136), and macrophages (ρ = 0.313, q = 0.0309). Conversely, FAM171A2 expression showed significant inverse correlations with several immune-cell scores, including mast cells (ρ = −0.450, q = 4.9 × 10^−4^), NK cells (ρ = −0.418, q = 0.0010), and central memory T cells (Tcm; ρ = −0.381, q = 0.0030). Although UPS was included in the subtype-specific survival analysis, only six UPS cases had matched immune-score data; thus, subtype-specific immune correlations for UPS were not deemed sufficiently reliable for inferential interpretation. Collectively, these findings indicate that the immune associations of FAM171A1 and FAM171A2 are histology-dependent, cautioning against the uniform extrapolation of pooled SARC immune correlations across sarcoma subtypes (Figure 10).

### 3.5. Gene Functional Network Analysis of FAM171A1 and FAM171A2

**Figure 11:**
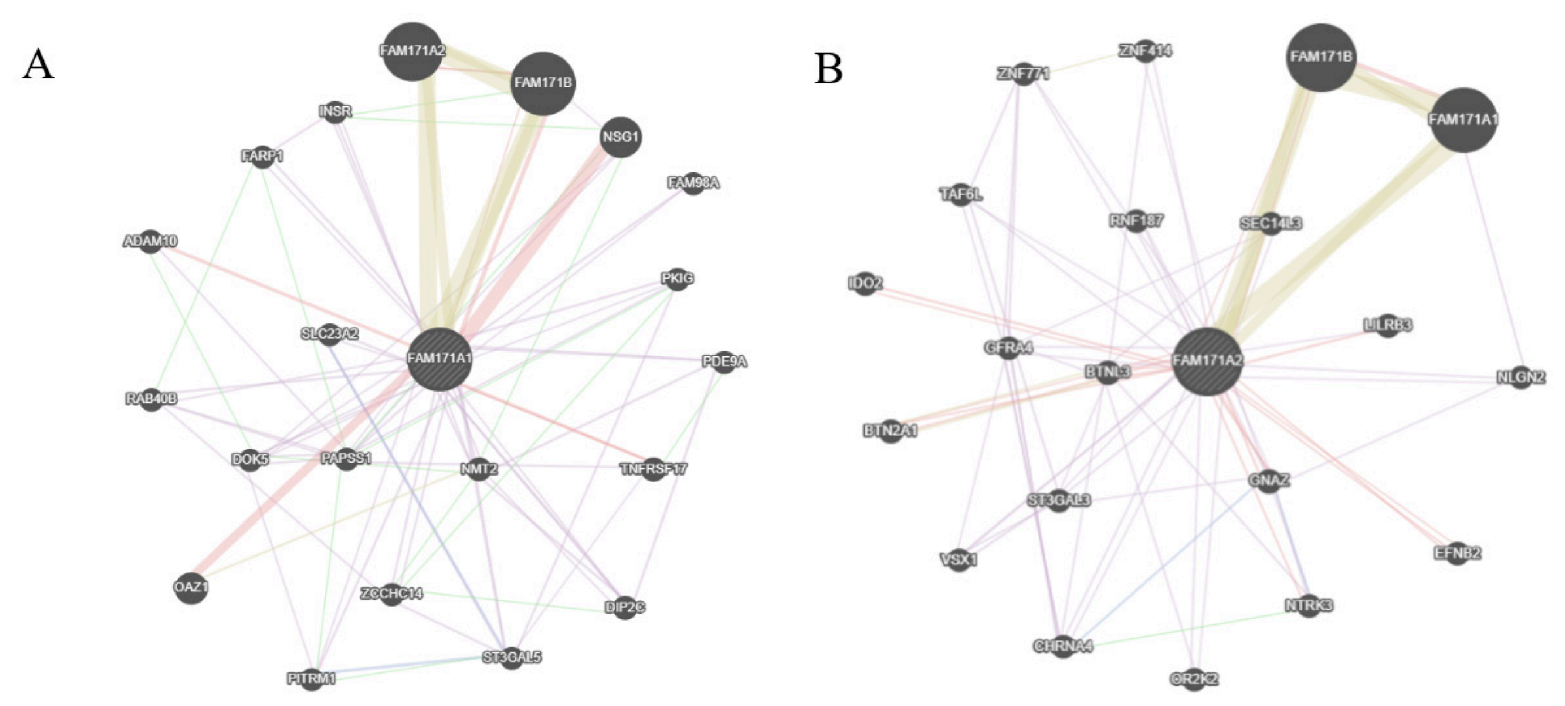
Gene functional interaction networks of FAM171A1 and FAM171A2 generated using GeneMANIA. (**A**) Functional interaction network of FAM171A1. (**B**) Functional interaction network of FAM171A2. Edges represent functional associations based on co-expression, physical interactions, genetic interactions, co-localization, pathway associations, and shared protein domains.

GeneMANIA analysis identified multiple genes functionally associated with FAM171A1 through co-expression, physical interactions, genetic interactions, and shared protein domains (Figure 11A; Table 1). Among the co-expressed genes, SLC23A2, INSR, DIP2C, FARP1, and RAB40B were identified as prominent co-expressed genes, whereas physical interaction analysis identified NSG1, OAZ1, ADAM10, FAM171B, and TNFRSF17 as major interacting partners. Shared protein domain analysis further revealed conserved domains with FAM171B and FAM171A2. Similarly, GeneMANIA analysis identified multiple genes functionally associated with FAM171A2 through co-expression, physical interactions, and shared protein domains (Figure 11B; Table 2). Among the co-expressed genes, ST3GAL3, GFRA4, CHRNA4, GNAZ, and NTRK3 were identified as prominent co-expressed genes, whereas physical interaction analysis identified FAM171B, GNAZ, BTNL3, BTN2A1, IDO2, LILRB3, EFNB2, and NTRK3 as major interacting partners. Shared protein domain analysis further revealed conserved domains with FAM171B and FAM171A1. These GeneMANIA-derived relationships represent database-supported functional associations and were considered exploratory; they do not establish direct molecular interactions or pathway regulation by FAM171A1 or FAM171A2.

### 3.6. Prediction of miRNAs Targeting FAM171A1 and FAM171A2

TargetScan analysis predicted multiple conserved miRNAs targeting FAM171A1, including hsa-miR-182-5p, hsa-miR-96-5p, hsa-miR-1271-5p, hsa-miR-153-3p, hsa-miR-124-3p, hsa-miR-181c-5p, hsa-miR-181a-5p, hsa-miR-4262, hsa-miR-181b-5p, hsa-miR-181d-5p, and hsa-miR-183-5p. Similarly, TargetScan predicted multiple conserved miRNAs targeting FAM171A2, including hsa-miR-6838-5p, hsa-miR-15b-5p, hsa-miR-497-5p, hsa-miR-424-5p, hsa-miR-195-5p, hsa-miR-16-5p, and hsa-miR-15a-5p. In silico prediction and correlation analysis of miRNAs associated with FAM171A1 and FAM171A2 were conducted using TargetScan, which identified several miRNAs with predicted binding sites in the transcripts of FAM171A1 and FAM171A2. Subsequently, ENCORI-based expression analysis was employed to investigate the relationships between these predicted miRNAs and their corresponding genes within the SARC cohort. Several miRNAs demonstrated statistically significant expression correlations with either FAM171A1 or FAM171A2. Notably, positive associations were observed, such as miR-181a-5p and miR-181b-5p with FAM171A1, and miR-424-5p with FAM171A2. These correlations indicate expression-level associations but do not confirm direct miRNA–target regulation. Therefore, the combined findings from TargetScan and ENCORI are interpreted as in silico candidate regulatory relationships that necessitate experimental validation.

### 3.7. miRNA–Target Co-Expression Analysis

**Figure 12:**
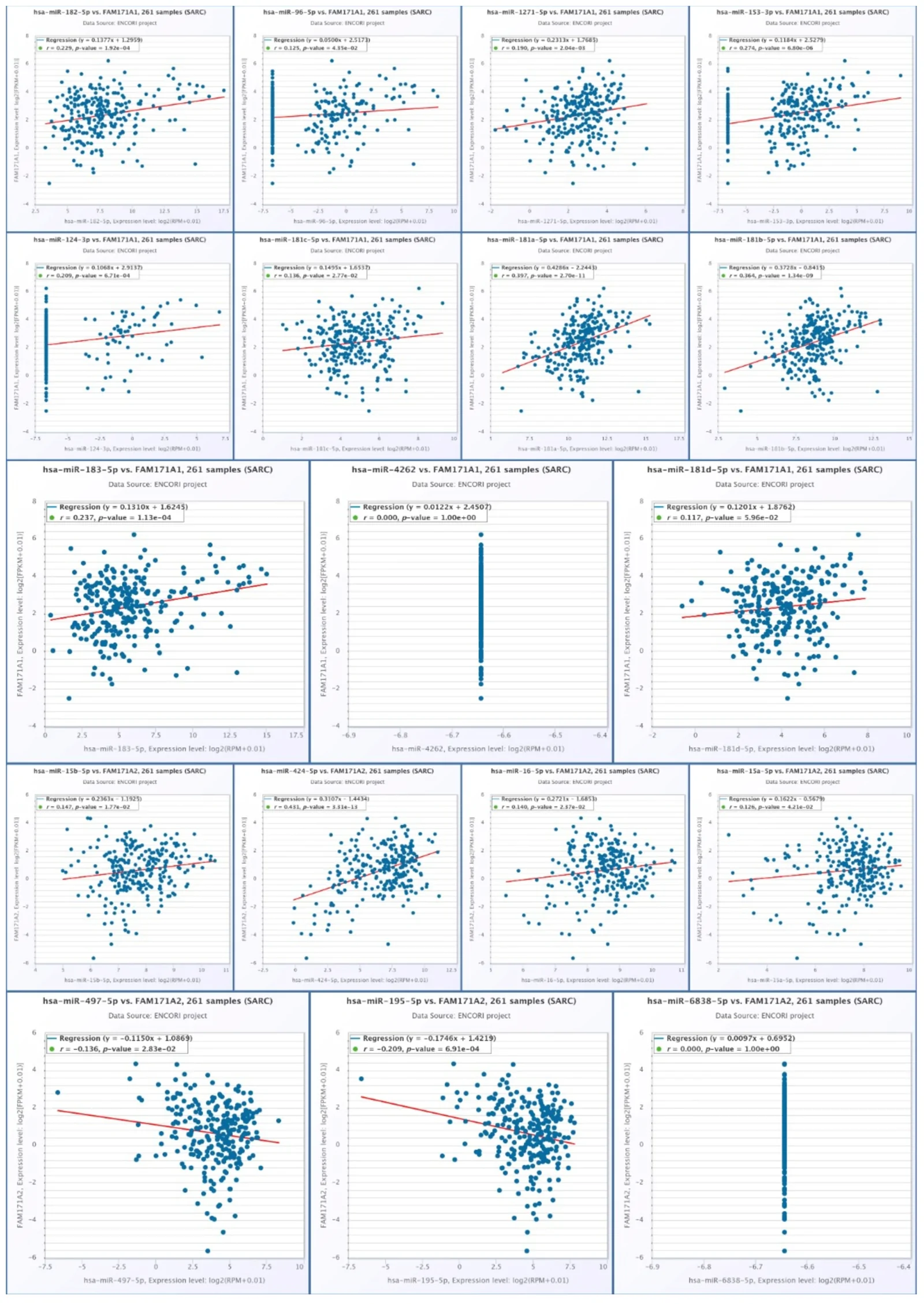
miRNA–target co-expression analysis of FAM171A1 and FAM171A2 in sarcoma using the ENCORI database. Correlation coefficients (*r*) and corresponding *p* values were obtained from the ENCORI platform.

Co-expression analysis in sarcoma tissues (n = 261) was performed using the ENCORI database. Several predicted miRNAs were significantly correlated with FAM171A1 expression (Figure 12). Significant positive correlations were observed for hsa-miR-182-5p (*r* = 0.229, *p* = 1.92 × 10^−4^), hsa-miR-96-5p (*r* = 0.125, *p* = 4.35 × 10^−2^), hsa-miR-1271-5p (*r* = 0.190, *p* = 2.04 × 10^−3^), hsa-miR-153-3p (*r* = 0.274, *p* = 6.80 × 10^−6^), hsa-miR-124-3p (*r* = 0.209, *p* = 6.71 × 10^−4^), hsa-miR-181c-5p (*r* = 0.136, *p* = 2.77 × 10^−2^), hsa-miR-181a-5p (*r* = 0.397, *p* = 2.70 × 10^−11^), hsa-miR-181b-5p (*r* = 0.364, *p* = 1.34 × 10^−9^), and hsa-miR-183-5p (*r* = 0.237, *p* = 1.13 × 10^−4^). In contrast, hsa-miR-4262 (*r* = 0.000, *p* = 1.00) and hsa-miR-181d-5p (*r* = 0.117, *p* = 5.96 × 10^−2^) showed no significant correlation with FAM171A1 expression (Figure 12). For FAM171A2, significant positive correlations were observed with hsa-miR-15b-5p (*r* = 0.147, *p* = 1.77 × 10^−2^), hsa-miR-424-5p (*r* = 0.431, *p* = 3.31 × 10^−13^), hsa-miR-16-5p (*r* = 0.140, *p* = 2.37 × 10^−2^), and hsa-miR-15a-5p (r = 0.126, *p* = 4.21 × 10^−2^), whereas hsa-miR-497-5p (*r* = −0.136, *p* = 2.83 × 10^−2^) and hsa-miR-195-5p (*r* = −0.209, *p* = 6.91 × 10^−4^) showed significant negative correlations. No significant association was observed for hsa-miR-6838-5p (*r* = 0.000, *p* = 1.00) (Figure 12). TargetScan results were regarded as computational predictions of potential miRNA-binding interactions. Correlations derived from ENCORI were utilized solely to evaluate expression-level associations and were not construed as evidence of direct miRNA-mediated regulation. Consequently, in the absence of functional assays, no predicted miRNA–target interaction was deemed experimentally validated.

## 4. Discussion

Sarcomas are highly heterogeneous malignancies with diverse biological behavior and generally poor clinical outcomes, highlighting the need for a better understanding of the molecular mechanisms underlying tumor progression and for the identification of novel biomarkers and therapeutic targets [4,26]. To address this gap, we performed an integrated bioinformatic analysis to evaluate the expression patterns, prognostic associations, immune infiltration, functional interaction networks and miRNA-related relationships of FAM171A1 and FAM171A2 in sarcoma. Analysis of gene expression revealed that the expression levels of FAM171A1 and FAM171A2 were significantly elevated in the pooled SARC cohort compared to the GEPIA3 normal-reference dataset. Furthermore, this increased expression was correlated with reduced overall survival. Functional network, immune infiltration, and miRNA-related analyses provided additional, hypothesis-generating context for the potential biological relevance of these genes in sarcoma. Collectively, these findings identify FAM171A1 and FAM171A2 as candidate genes showing histology-dependent expression, survival, and immune associations in sarcoma and provide a rationale for their further experimental and clinical investigation. The current findings should be regarded as generating hypotheses rather than establishing either gene as a clinically validated prognostic biomarker. A significant outcome of this study is the variable expression and clinical associations of FAM171A1 and FAM171A2 across different sarcoma histologies. Analysis stratified by histology revealed significant differences in the expression of both genes among major sarcoma subtypes. Notably, FAM171A1 exhibited marked heterogeneity, with relatively higher expression in MPNST, synovial sarcoma, and DDLPS, and lower expression in LMS. Similarly, FAM171A2 showed significant subtype-dependent variation, with increased expression in synovial sarcoma and DDLPS. Given the small MPNST (n = 9) and synovial sarcoma (n = 10) groups, observations involving these subtypes should be regarded as exploratory. These findings are crucial because sarcoma consists of biologically distinct entities characterized by diverse cellular origins, genomic alterations, transcriptional programs, and clinical behaviors. Therefore, the increased expression observed in the pooled SARC cohort should not be regarded as a uniform molecular characteristic of all sarcoma histologies. Instead, our results indicate that the involvement of FAM171A1 and FAM171A2 may be contingent upon the histological context.

The findings regarding tumor–normal expression necessitate careful interpretation. The normal reference samples used in the GEPIA3 analysis incorporate GTEx tissues, while sarcomas originate from various anatomical and mesenchymal tissue contexts. As a result, the elevated expression of FAM171A1 and FAM171A2 observed in the pooled TCGA-SARC compared to the normal-reference cannot be solely attributed to tumor-specific dysregulation, as variations in tissue composition may influence the observed expression patterns. To address this, we analyzed an independent non-TCGA RNA-sequencing cohort (GSE198568) that includes paired soft-tissue sarcoma and adjacent normal tissues. Both genes exhibited higher expression in tumors, with FAM171A2 showing increased expression in all four matched tumor–normal pairs; however, these differences did not achieve statistical significance due to the limited number of available pairs. Consequently, this independent dataset offers supportive directional evidence rather than definitive external validation. We therefore examined a second independent cohort (GSE159659) comprising 15 patients with retroperitoneal liposarcoma and normal adipose tissue obtained from the same anatomical site. In this cohort, FAM171A2 upregulation was reproduced, most evidently in DDLPS, although the effect size was modest and did not remain significant after genome-wide correction, whereas FAM171A1 showed no tumor–normal difference. Because GSE159659 is restricted to liposarcoma and uses adipose tissue as the normal comparator, these results cannot be generalized to other sarcoma subtypes. Larger, multi-subtype cohorts with histologically and anatomically matched normal tissues are needed to determine whether these differences consistently represent tumor-associated dysregulation.

FAM171A1 has been implicated in the regulation of actin cytoskeleton dynamics and cell morphology, processes closely associated with tumor cell motility and invasion. Previous studies have demonstrated that increased FAM171A1 expression disrupts actin stress fiber organization and promotes invasive tumor growth [7]. Elevated FAM171A1 expression has also been reported in several malignancies, including colon cancer, OSCC, and TNBC [8,11,12,13]. In agreement with these findings, FAM171A1 expression was higher in the pooled SARC cohort than in the normal-reference dataset; however, this difference was not reproduced in the matched liposarcoma cohort (GSE159659), indicating that FAM171A1 upregulation may be histology-dependent or partly attributable to differences in the normal reference tissues. Furthermore, RNA expression data from HPA demonstrated detectable FAM171A1 expression across multiple sarcoma-derived cell lines, supporting its relevance for subsequent experimental investigation in sarcoma models. Compared with FAM171A1, the biological role of FAM171A2 remains considerably less characterized. Previous studies have mainly focused on its neuronal functions, particularly its involvement in synaptic signaling, vesicle trafficking, and the propagation of pathological α-synuclein, whereas evidence regarding its role in cancer remains limited [14,15]. A recent bioinformatic study reported increased FAM171A2 expression in ovarian cancer but not in uterine corpus endometrial carcinoma, suggesting that its expression may be cancer type-dependent [9]. In the present study, FAM171A2 was also significantly upregulated in sarcoma tissues, and its expression was consistently detected across multiple sarcoma-derived cell lines. This upregulation was also reproduced in the matched GSE159659 cohort, most evidently in DDLPS. These findings extend the current knowledge by identifying FAM171A2 as another FAM171 family member that is overexpressed in sarcoma. Taken together, FAM171A2 showed the more consistent tumor-associated expression pattern across the pooled and independent cohorts, whereas evidence for FAM171A1 upregulation was less consistent. The detection of both genes in sarcoma-derived cell lines supports their suitability for subsequent experimental investigation in sarcoma models.

Patients with sarcoma frequently experience unfavorable clinical outcomes due to tumor heterogeneity, recurrence, and metastatic progression, emphasizing the need for reliable prognostic biomarkers [4,27]. The prognostic relevance of FAM171A1 has been reported in several malignancies. Elevated FAM171A1 expression has been associated with poor prognosis in CC [8], as well as with increased tumor size and recurrence in OTSCC [13]. In breast cancer, particularly TNBC, FAM171A1 has been identified as a hub gene and proposed as a potential biomarker associated with tumor aggressiveness based on integrated transcriptomic analyses [10,11]. Consistent with these findings, elevated FAM171A1 expression was associated with poorer OS in patients with sarcoma, both in the exploratory Kaplan–Meier analysis and in the main continuous-variable multivariable Cox analysis, whereas no significant association was observed with RFS. In contrast, the prognostic significance of FAM171A2 has been only rarely investigated in cancer. A recent bioinformatic study of gynecological malignancies reported no significant association between FAM171A2 expression and OS [9]. In our study, however, elevated FAM171A2 expression was associated with poorer OS in both analyses, whereas no significant association was observed with RFS. To the best of our knowledge, this is the first study to report a prognostic association of FAM171A2 expression in sarcoma. Collectively, these findings indicate prognostic associations of FAM171A1 and FAM171A2 expression in sarcoma; however, their clinical biomarker utility cannot be established from the present dataset and requires independent validation. The histology-dependent nature of these associations was further corroborated by the subtype-specific survival analyses. While elevated expression of FAM171A1 and FAM171A2 correlated with poorer overall survival in the pooled SARC analysis, this relationship was not consistently observed across individual histological subtypes. Neither gene demonstrated a statistically significant association with overall survival in LMS or DDLPS. Conversely, high expression levels of both FAM171A1 and FAM171A2 were linked to significantly poorer overall survival in UPS, with hazard ratios of 3.66 and 3.98, respectively. Notably, these associations remained significant after adjusting for multiple subtype-specific survival comparisons. These findings imply that the prognostic associations of the FAM171 family in sarcoma may be contingent on histology, with a potential association in UPS. However, the relatively small number of survival events within the UPS subgroup and the consequent wide confidence intervals necessitate caution. Thus, these findings should be considered exploratory evidence of a subtype-specific prognostic association rather than definitive proof that FAM171A1 or FAM171A2 serves as an established prognostic biomarker in UPS. In the present study, these findings are interpreted as prognostic associations rather than established prognostic biomarkers.

The subtype-stratified survival findings should be interpreted with particular caution. Although the UPS analyses identified potentially relevant associations, histology-specific sample sizes and numbers of events were substantially smaller than in the pooled cohort, resulting in wider uncertainty around the estimates. Accordingly, these subtype-specific survival associations should be regarded as exploratory and require confirmation in independent cohorts with adequate sample sizes and events.

Because the initial Kaplan–Meier analyses relied on a data-driven cutoff that may overestimate prognostic associations, the histology-stratified multivariable Cox regression models, in which gene expression was treated as a continuous variable, were considered the main survival analysis. In these models, FAM171A1 and FAM171A2 expression was treated as a continuous variable, and the analyses were adjusted for age and sex, allowing baseline hazards to vary across histological subtypes. The results indicated that higher expression levels of both genes were significantly associated with poorer overall survival. These associations persisted in sensitivity analyses that additionally accounted for radiotherapy and residual tumor status. Consequently, the relationship between FAM171A1/FAM171A2 expression and survival does not appear to be solely attributable to differences in histological composition or the available clinical covariates. However, these findings should be regarded as adjusted prognostic associations rather than definitive evidence of clinical biomarkers. Furthermore, proportional-hazards diagnostics revealed modest time dependence in the gene effects, suggesting that caution is warranted when interpreting the estimated hazard ratios as constant effects throughout follow-up. Independent validation in adequately powered cohorts with comprehensive clinicopathological data is necessary before the prognostic utility of either gene can be confirmed. Notably, the independent GEO cohorts analyzed in this study (GSE198568 and GSE159659) provided expression data only and did not include survival information; therefore, they support the tumor–normal expression findings but do not provide external validation of the prognostic associations observed in TCGA-SARC.

Tumor progression is closely associated with the tumor microenvironment and immune cell infiltration, both of which influence tumor behavior, therapeutic response, and clinical outcome [1,4,27]. Although sarcomas have traditionally been considered relatively immune-cold tumors, accumulating evidence indicates that their immune landscape is highly heterogeneous across histological subtypes [1]. This heterogeneity is consistent with the comprehensive molecular characterization of adult soft tissue sarcomas by the Cancer Genome Atlas Research Network, which demonstrated substantial genomic and regulatory diversity across sarcoma types and showed that the inferred immune microenvironment was associated with clinical outcome, highlighting the potential relevance of immune context in sarcoma [28]. More recent studies have further emphasized that the sarcoma microenvironment comprises distinct transcriptionally defined cell states and multicellular ecosystems that are associated with patient outcomes and, in specific cellular contexts, may also predict response to immune checkpoint inhibition [29]. Accordingly, characterization of tumor-infiltrating immune cells has become increasingly important for understanding the biological heterogeneity of sarcoma.

To date, the relationship between FAM171A1 and FAM171A2 expression and immune cell infiltration has not been investigated in sarcoma. In the present study, FAM171A1 expression was negatively correlated with tumor purity, CD8^+^ T cell infiltration, and dendritic cell infiltration, whereas FAM171A2 expression showed significant negative correlations with CD8^+^ T cells, CD4^+^ T cells, and dendritic cells. TIMER3 analysis further indicated that the associations between FAM171A1/FAM171A2 expression and Treg infiltration were algorithm-dependent, with significant correlations detected by some immune deconvolution methods but not by others. These differences likely reflect methodological variation in immune cell estimation among the algorithms. Therefore, these findings warrant further validation. The negative associations observed with CD8^+^ T cell infiltration are of particular interest, as CD8^+^ T cells are key mediators of antitumor immunity, and increased CD8^+^ T cell infiltration has been associated with improved survival and therapeutic response in several malignancies, including sarcoma [1,4]. Likewise, CD4^+^ T cells play important roles in coordinating antitumor immune responses [1]. Dendritic cells are key mediators of antigen presentation and antitumor immunity [30]. In this context, the broader sarcoma literature indicates that the relationship between immune-cell composition and clinical behavior is not uniform across histologies. Recine et al. emphasized that the tumor immune microenvironment of sarcomas is shaped by complex interactions among malignant cells, immune populations, stromal components, and extracellular matrix, with immune-related features potentially contributing to both prognosis and therapeutic response [31]. Moreover, alterations in antigen-processing and presentation machinery have been documented in sarcomas and may represent an additional mechanism influencing tumor–immune interactions [32]. Therefore, the observed negative correlations with these immune cell populations suggest that FAM171A1 and FAM171A2 may be associated with the immune microenvironment of sarcoma. Interestingly, Hu et al. reported that FAM171B, another member of the FAM171 family, promotes CCL2-mediated tumor-associated macrophage infiltration in bladder cancer [33]. Although the biological functions of FAM171 family members may differ across tumor types, this finding provides indirect evidence that members of this gene family may participate in tumor–immune interactions. Taken together, these findings suggest that FAM171A1 and FAM171A2 expression is associated with immune infiltration patterns in sarcoma; however, because these associations are correlative and derived from computational estimates, they do not indicate that either gene regulates immune-cell recruitment or antitumor immunity.

Subtype-specific immune analyses further elucidated that the associations between FAM171A1/FAM171A2 expression and the tumor immune microenvironment vary according to sarcoma histology. This observation is in line with previous multi-platform and microenvironment-focused studies demonstrating that sarcomas comprise biologically distinct histological entities with different molecular and immune contexts [28,29,31]. The most notable pattern was identified in DDLPS, where elevated expression of both genes, particularly FAM171A2, correlated with reduced infiltration scores for several immune-cell populations. FAM171A2 exhibited inverse correlations with cytotoxic cells, total T cells, CD8 T cells, dendritic cells, macrophages, neutrophils, and B cells, while FAM171A1 was also inversely associated with multiple immune populations, including activated dendritic cells, cytotoxic cells, T cells, and CD8 T cells. This coordinated inverse relationship may suggest that increased FAM171A expression in DDLPS is linked to a relatively immune-depleted tumor microenvironment. However, as these analyses are correlation-based, they do not establish whether FAM171A1 or FAM171A2 directly regulates immune-cell recruitment or function. Furthermore, the findings of Renne et al. indicate that alterations in antigen-processing and presentation machinery can differ among sarcoma histologies, reinforcing the concept that immune recognition and tumor–immune interactions may be biologically heterogeneous across sarcoma subtypes [32]. Although our study did not directly evaluate antigen-processing or presentation pathways, this provides a relevant biological context for interpreting the subtype-specific immune associations observed for FAM171A1 and FAM171A2. In LMS, a distinct immune-association pattern emerged. FAM171A1 expression was positively associated with TFH cells, NK CD56dim cells, and macrophages, whereas FAM171A2 predominantly showed inverse relationships with several immune populations, including mast cells, NK cells, central memory T cells, dendritic cells, and cytotoxic cells. The contrasting patterns observed between LMS and DDLPS, as well as between FAM171A1 and FAM171A2 within LMS, further indicate that the immune relevance of these genes cannot be generalized across the pooled SARC cohort. Instead, their associations with the immune microenvironment appear to depend on both histological context and the individual FAM171 family member. This interpretation is consistent with evidence that sarcoma microenvironmental ecosystems can differ substantially in their cellular composition and clinical associations, supporting a histology-aware rather than uniformly pooled interpretation of immune-related findings [29,31]. Collectively, the subtype-stratified analyses significantly refine the interpretation of the pooled SARC findings. Although the pooled analyses identified FAM171A1 and FAM171A2 as genes associated with sarcoma expression, overall survival, and immune infiltration, the current histology-specific analyses demonstrate that these relationships vary considerably among sarcoma entities. A preliminary prognostic association was observed in UPS, whereas the strongest subtype-specific immune associations were observed in DDLPS and, to a different extent, LMS. Thus, FAM171A1 and FAM171A2 should not currently be considered universal prognostic biomarkers across sarcoma. Rather, the present findings support a histology-aware model in which their biological and potential prognostic relevance may differ among individual sarcoma subtypes. Validation in independent, adequately powered histology-specific cohorts and multivariable analyses incorporating established clinicopathological prognostic factors will be necessary before subtype-specific clinical utility can be established.

The comparative analysis reveals that the shared membership of FAM171A1 and FAM171A2 within the FAM171 family should not be construed as indicative of functional equivalence. Although both genes demonstrated associations with overall survival, their expression distributions and immune associations varied across histological subtypes. This distinction was particularly evident in LMS, where FAM171A1 exhibited positive associations with several immune-cell populations, whereas FAM171A2 predominantly showed inverse associations. Therefore, the significance of analyzing these genes in parallel lies not in presuming a common biological function, but in identifying potentially convergent and divergent patterns among related family members. Whether these differences reflect distinct molecular functions or indirect context-dependent associations cannot be ascertained from the current computational analyses and necessitate experimental investigation.

GeneMANIA integrates diverse sources of functional genomic and proteomic data to identify functionally associated genes and biological interaction networks, thereby offering a valuable framework for exploring potential gene functions [23]. In this study, GeneMANIA analysis provided an exploratory, database-derived view of the network context of FAM171A1 and FAM171A2 by identifying genes associated through co-expression, physical interactions, genetic interactions, and shared protein domains. For FAM171A1, the primary co-expressed genes included SLC23A2, INSR, DIP2C, FARP1, and RAB40B, while physical interactions were identified with NSG1, OAZ1, ADAM10, FAM171B, and TNFRSF17. These genes are implicated in biological processes related to cytoskeletal organization, intracellular trafficking, receptor signaling, cell adhesion, and transmembrane transport, suggesting that FAM171A1 may be involved in cellular pathways pertinent to tumor progression [34,35,36,37,38]. Although these predicted functional associations do not establish direct biological interactions, they align with previous evidence indicating that FAM171A1 contributes to cytoskeletal organization and tumor invasion [7]. In contrast to FAM171A1, the biological functions of FAM171A2 are less well understood. GeneMANIA analysis identified ST3GAL3, GFRA4, CHRNA4, GNAZ, and NTRK3 as major co-expressed genes, while physical interactions were predicted with FAM171B, GNAZ, BTNL3, BTN2A1, IDO2, LILRB3, EFNB2, and NTRK3. Collectively, these genes are associated with biological processes related to receptor-mediated signaling, neuronal communication, immune regulation, protein glycosylation, cell adhesion, and cell migration [39,40,41,42,43,44]. The GeneMANIA analysis provided an exploratory perspective on the functional association networks surrounding FAM171A1 and FAM171A2. The identified relationships encompassed co-expression, database-derived physical and genetic interactions, and shared protein domains. These network connections may assist in prioritizing candidate genes and biological processes for further investigation; however, they should not be interpreted as evidence that FAM171A1 or FAM171A2 directly regulates the associated pathways in sarcoma. Specifically, associations with genes involved in tumor-related signaling or immune processes do not establish pathway activation, causal regulation, or a mechanistic role for either FAM171 family member. Consequently, the GeneMANIA findings are considered hypothesis-generating and provide a framework for future functional studies rather than mechanistic evidence. Experimental perturbation of FAM171A1 and FAM171A2 in histologically defined sarcoma models, followed by assessment of predicted network partners and downstream cellular phenotypes, will be necessary to determine whether any of these computationally identified associations have functional relevance.

MicroRNAs are important post-transcriptional regulators of gene expression and have emerged as critical modulators of sarcoma development and progression through the regulation of cell proliferation, differentiation, invasion, metastasis, and other cancer-related processes [5]. In this study, TargetScan predicted multiple conserved miRNAs targeting FAM171A1 and FAM171A2. Subsequent co-expression analysis using the ENCORI database demonstrated significant correlations between several predicted miRNAs and their respective target genes. For FAM171A1, members of the miR-183/96/182 cluster (miR-183-5p, miR-96-5p, and miR-182-5p) and the miR-181 family (miR-181a-5p, miR-181b-5p, and miR-181c-5p) exhibited significant positive correlations with gene expression. The miR-183/96/182 cluster, also known as miR-183C, is an evolutionarily conserved miRNA cluster that plays important roles in normal sensory organ development and function [45]. Moreover, accumulating evidence has shown that dysregulation of this miRNA cluster contributes to key biological processes involved in tumor progression, including cell proliferation, migration, invasion, and angiogenesis [45,46,47]. Likewise, members of the miR-181 family have been reported to regulate multiple cancer-related processes, including proliferation, apoptosis, angiogenesis, autophagy, and drug resistance, with context-dependent oncogenic or tumor-suppressive functions [48,49]. In addition, miR-124-3p and miR-153-3p were also positively correlated with FAM171A1 expression, expanding the predicted miRNA profile associated with FAM171A1 in sarcoma. Compared with FAM171A1, FAM171A2 exhibited a distinct miRNA correlation pattern in sarcoma. Positive correlations were observed with miR-15a-5p, miR-15b-5p, miR-16-5p, and miR-424-5p, whereas miR-195-5p and miR-497-5p showed negative correlations. Notably, these miRNAs belong to the miR-15/16/195/424/497 family, whose members share a conserved seed sequence and have been reported to regulate multiple target genes involved in cell-cycle regulation, apoptosis, and other cancer-related processes [50,51,52,53]. Among these family members, miR-497 has been reported to be downregulated in multiple malignancies, including osteosarcoma, and its restoration suppresses tumor cell proliferation, migration, and invasion, further supporting its tumor-suppressive role [53,54,55]. Overall, these findings indicate that FAM171A1 and FAM171A2 are associated with distinct miRNA expression patterns in sarcoma, providing a foundation for future studies investigating their post-transcriptional regulation and biological functions. The miRNA analyses conducted in this study should be regarded as hypothesis-generating rather than providing mechanistic evidence. TargetScan identifies potential miRNA-binding relationships based on sequence, while ENCORI correlation analysis assesses expression-level associations within the analyzed cohort. Neither method demonstrates direct miRNA-mediated regulation of FAM171A1 or FAM171A2. Furthermore, several predicted miRNAs, such as miR-181a-5p and miR-181b-5p for FAM171A1 and miR-424-5p for FAM171A2, exhibited positive rather than inverse correlations with their predicted target genes. Because canonical miRNA-mediated repression would be expected to produce inverse miRNA–target correlations, these positive correlations do not support direct miRNA-mediated repression of FAM171A1 or FAM171A2. Although miRNA–mRNA interactions in biological systems may be affected by indirect regulatory networks, cell-type composition, feedback mechanisms, and other context-dependent processes, the observed positive correlations do not validate the predicted TargetScan interactions. Consequently, the current findings suggest candidate miRNA–FAM171 relationships for future research but do not confirm functional miRNA-mediated regulation. Experimental studies are necessary to ascertain whether any of these predicted interactions are biologically functional. Such validation should involve modulation of candidate miRNAs using mimic and inhibitor approaches, followed by evaluation of FAM171A1 or FAM171A2 expression at both mRNA and protein levels. For candidates showing consistent regulatory effects, wild-type and mutated 3′UTR luciferase reporter assays would be essential to establish direct binding to the predicted target site. Until these experiments are conducted, the miRNA findings of this study should be considered strictly as computationally predicted and correlation-supported hypothesis.

The current findings should be interpreted in light of the exploratory and computational nature of the study. While the expression of FAM171A1 and FAM171A2 was linked to clinical outcomes and various features of the sarcoma molecular and immune landscape, these associations do not confirm a direct functional role for either gene in tumor progression, invasion, or immune regulation. Similarly, the regulatory and network analyses suggest potential biological relationships rather than experimentally verified mechanisms. To establish the functional relevance of FAM171A1 and FAM171A2, it is necessary to validate their expression at both the mRNA and protein levels in histologically defined sarcoma models and appropriate controls, followed by loss- and gain-of-function experiments. Evaluating proliferation, clonogenic capacity, migration, invasion, apoptosis, and other phenotype-specific responses would help ascertain whether manipulation of either gene directly influences sarcoma cell behavior. Subsequent mechanistic studies should then concentrate on the regulatory or signaling relationships most strongly supported by these functional experiments. Until such validation is achieved, the current findings should be regarded as hypothesis-generating evidence that prioritizes FAM171A1 and FAM171A2 for further investigation, rather than demonstrating their functional involvement in sarcoma.

### Limitations

This study has several limitations. First, all analyses were performed using publicly available datasets and bioinformatic platforms without experimental validation. Therefore, the findings should be interpreted as hypothesis-generating rather than conclusive evidence. Second, sarcoma represents a highly heterogeneous group of malignancies comprising multiple histological subtypes with distinct molecular characteristics, whereas most analyses were performed using the combined TCGA-SARC cohort. While histology-stratified analyses were employed to address the heterogeneity within the pooled SARC cohort, the sample size significantly diminished post-stratification. This limitation was particularly pertinent to survival and immune analyses, where the number of events or matched immune-profiled samples was inadequate for a reliable evaluation of several less common histological subtypes. For instance, the immune analysis specific to the UPS subtype was not interpreted due to the availability of matched immune-infiltration data for only six tumors. Additionally, the subtype-specific survival analyses were exploratory and based on univariable models; thus, the associations observed, especially those identified in UPS, necessitate validation in larger independent cohorts and confirmation through multivariable models that incorporate established clinicopathological prognostic factors. In addition, the initial pooled Kaplan–Meier analyses used an automatically optimized cutoff, which may overestimate prognostic associations; these results were therefore considered exploratory, and the continuous-variable multivariable Cox analyses were regarded as the main survival analysis. Another limitation pertains to external validation. The GSE198568 cohort included only four paired samples, and the larger matched GSE159659 cohort was restricted to retroperitoneal liposarcoma with adipose tissue as the normal comparator; moreover, FAM171A1 upregulation was not reproduced in this cohort. Moreover, an appropriate independent non-TCGA sarcoma cohort, which could provide reliable measurements of both FAM171A1 and FAM171A2 along with sufficiently detailed individual-level overall survival data, was unavailable for direct prognostic replication. Consequently, although associations with overall survival remained significant in multivariable and sensitivity analyses within TCGA-SARC, independent prognostic validation is still required. Furthermore, the use of GTEx-derived normal reference tissues in the pooled expression comparison introduces potential tissue-of-origin confounding, thereby limiting the interpretation of these differences as tumor-specific dysregulation.

The miRNA analyses conducted in this study were exclusively computational, lacking support from functional experiments. Predictions from TargetScan and expression correlations from ENCORI do not confirm direct miRNA–target regulation, especially for predicted miRNAs that exhibit positive correlations with FAM171A1 or FAM171A2. Consequently, these results should be regarded as hypothesis-generating until they are validated through miRNA perturbation experiments and direct 3′UTR binding assays.

Similarly, the immune-infiltration analyses were based on immune-cell abundances estimated computationally from bulk tumor transcriptomes rather than on direct measurements such as immunohistochemistry, flow cytometry, or single-cell profiling. The estimated associations, particularly those involving Treg infiltration, varied according to the deconvolution algorithm used, and all immune analyses were correlative. Consequently, these findings cannot establish whether FAM171A1 or FAM171A2 influences immune-cell recruitment, function, or the antitumor immune response, and they require validation using direct tissue-based immune profiling and functional studies.

Moreover, the GeneMANIA network analysis relies on functional associations derived from databases and does not provide experimental evidence of direct interactions, pathway regulation, or mechanistic activity of FAM171A1 or FAM171A2 in sarcoma.

The primary limitation of this study is the lack of experimental functional validation. The analyses were primarily based on publicly available transcriptomic, clinical, immune-infiltration, and database-derived network data. As a result, associations between FAM171A1/FAM171A2 expression and survival, immune features, predicted miRNA relationships, or functional networks cannot establish causality or direct molecular mechanisms. Neither gene was experimentally manipulated in sarcoma models, and effects on proliferation, migration, invasion, apoptosis, or other tumor-related phenotypes were not evaluated. Additionally, differential expression was not independently confirmed at both the mRNA and protein levels in a sufficiently powered experimental cohort. These limitations prevent conclusions regarding direct functional involvement or therapeutic actionability and should be addressed in future experimental studies.

In addition, the histology-stratified analyses included small subgroups, particularly MPNST and synovial sarcoma. These factors limit the precision and generalizability of subtype-specific estimates.

Finally, although multiple complementary bioinformatic approaches were integrated, further in vitro, in vivo, and clinical studies are required to validate the biological functions, regulatory mechanisms, and clinical significance of FAM171A1 and FAM171A2 in sarcoma.

## 5. Conclusions

This comparative multi-platform bioinformatic study illustrates that FAM171A1 and FAM171A2 display clinically associated yet non-uniform expression, survival, and immune-associated patterns across sarcoma. Analyses stratified by histology revealed significant subtype-dependent heterogeneity, suggesting that findings from the pooled SARC cohort should not be uniformly generalized across all sarcoma entities. Tumor-associated upregulation of FAM171A2 was reproduced in an independent matched liposarcoma cohort, whereas that of FAM171A1 was not. Notably, the associations of both genes with overall survival remained significant after adjusting for available clinical covariates and histological stratification, although their prognostic relevance necessitates confirmation in independent, adequately powered cohorts. The two FAM171 family members also exhibited distinct immune-association patterns across histological contexts, supporting their comparative evaluation while cautioning against assuming functional equivalence. GeneMANIA and miRNA analyses identified candidate network and regulatory relationships; however, these findings are computational and hypothesis-generating and should not be construed as evidence of direct molecular mechanisms. In summary, FAM171A1 and FAM171A2 emerge as candidate genes with distinct, histology-dependent clinical and biological associations in sarcoma. Their functional roles, mechanistic significance, and potential clinical utility require validation through independent clinical cohorts and targeted experimental studies. These findings should be considered exploratory and hypothesis-generating, particularly for histology-specific survival and immune-associated analyses, and should not be interpreted as establishing FAM171A1 or FAM171A2 as validated prognostic biomarkers.

## Figures and Tables

**Table 1:** Functionally associated genes and interaction types of FAM171A1.

Gene Symbol	Protein Name	Weight	Interaction Type
DIP2C	Disco interacting protein 2 homolog C	0.02002864	Co-expression
NMT2	N-myristoyltransferase 2	0.018383993	Co-expression
ZCCHC14	Zinc finger CCHC-type containing 14	0.012182159	Co-expression
FARP1	FERM, ARH/RhoGEF and pleckstrin domain protein 1	0.019724527	Co-expression
PDE9A	Phosphodiesterase 9A	0.017610738	Co-expression
PITRM1	Pitrilysin metallopeptidase 1	0.018139	Co-expression
PAPSS1	3′ phosphoadenosine 5′ phosphosulfate synthase 1	0.0049765804	Co-expression
DOK5	Docking protein 5	0.008092642	Co-expression
RAB40B	RAB40B, member RAS oncogene family	0.013648064	Co-expression
ST3GAL5	ST3 beta-galactoside alpha-2,3-sialyltransferase 5	0.011325117	Co-expression
SLC23A2	Solute carrier family 23 member 2	0.024824912	Co-expression
INSR	Insulin receptor	0.022508478	Co-expression
NSG1	Neuronal vesicle trafficking associated 1	0.010943811	Co-expression
PKIG	cAMP-dependent protein kinase inhibitor gamma	0.007910139	Co-expression
FAM98A	Family with sequence similarity 98 member A	0.0094463695	Co-expression
NSG1	Neuronal vesicle trafficking associated 1	0.0005111266	Genetic interaction
PAPSS1	3′ phosphoadenosine 5′phosphosulfate synthase 1	0.00043798093	Genetic interaction
NSG1	Neuronal vesicle trafficking associated 1	0.70301753	Physical interaction
OAZ1	Ornithine decarboxylase antizyme 1	0.41801673	Physical interaction
ADAM10	ADAM metallopeptidase domain 10	0.11062091	Physical interaction
FAM171B	Family with sequence similarity 171 member B	0.3220938	Physical interaction
TNFRSF17	TNF receptor superfamily member 17	0.23914285	Physical interaction
FAM171B	Family with sequence similarity 171 member B	0.5	Shared protein domain
FAM171A2	Family with sequence similarity 171 member A2	0.5	Shared protein domain

**Table 2:** Functionally associated genes and interaction types of FAM171A2.

Gene Symbol	Protein Name	Weight	Interaction Type
ST3GAL3	ST3 beta-galactoside alpha-2,3-sialyltransferase 3	0.007199702	Co-expression
RNF187	Ring finger protein 187	0.0067065656	Co-expression
SEC14L3	SEC14 like lipid binding 3	0.007042333	Co-expression
ZNF771	Zinc finger protein 771	0.0064050346	Co-expression
GFRA4	GDNF family receptor alpha 4	0.0067527597	Co-expression
CHRNA4	Cholinergic receptor nicotinic alpha 4 subunit	0.0070113097	Co-expression
OR2K2	Olfactory receptor family 2 subfamily K member 2	0.006524924	Co-expression
VSX1	Visual system homeobox 1	0.0064650513	Co-expression
TAF6L	TATA-box binding protein associated factor 6 like	0.006920082	Co-expression
ZNF414	Zinc finger protein 414	0.007093599	Co-expression
NLGN2	Neuroligin 2	0.006242274	Co-expression
GNAZ	G protein subunit alpha z	0.00068169413	Co-expression
NTRK3	Neurotrophic receptor tyrosine kinase 3	0.0033078608	Co-expression
FAM171B	Family with sequence similarity 171 member B	0.09908232	Physical interaction
GNAZ	G protein subunit alpha z	0.04914978	Physical interaction
BTNL3	Butyrophilin like 3	0.10238713	Physical interaction
BTN2A1	Butyrophilin subfamily 2 member A1	0.05337842	Physical interaction
IDO2	Indoleamine 2,3-dioxygenase 2	0.08535894	Physical interaction
LILRB3	Leukocyte immunoglobulin like receptor B3	0.0899751	Physical interaction
EFNB2	Ephrin B2	0.030111043	Physical interaction
NTRK3	Neurotrophic receptor tyrosine kinase 3	0.042523153	Physical interaction
FAM171B	Family with sequence similarity 171 member B	0.5	Shared protein domain
FAM171A1	Family with sequence similarity 171 member A1	0.5	Shared protein domain

## Data Availability

The data analyzed in this study are publicly available from TCGA (via GEPIA3, LinkedOmics, Kaplan–Meier Plotter, TIMER/TIMER3, and ENCORI), the Human Protein Atlas, and the Gene Expression Omnibus under accession numbers GSE198568 and GSE159659.

## Abbreviations

The following abbreviations are used in this manuscript:

AUC	Area under the receiver operating characteristic curve
CI	Confidence interval
CPM	Counts per million
DDLPS	Dedifferentiated liposarcoma
ENCORI	Encyclopedia of RNA interactomes
FAM171	Family with sequence similarity 171
FAM171A1	Family with sequence similarity 171 member A1
FAM171A2	Family with sequence similarity 171 member A2
FDR	False discovery rate
GEO	Gene Expression Omnibus
GEPIA3	Gene expression profiling interactive analysis 3
GTEx	Genotype-tissue expression project
HPA	Human protein atlas
HR	Hazard ratio
KM Plotter	Kaplan–Meier plotter
LMS	Leiomyosarcoma
MFS	Myxofibrosarcoma
miRNA	MicroRNA
MPNST	Malignant peripheral nerve sheath tumor
nTPM	Normalized transcripts per million
OS	Overall survival
OTSCC	Oral tongue squamous cell carcinoma
RFS	Relapse-free survival
RSEM	RNA-seq by expectation maximization
SARC	Sarcoma
SS	Synovial sarcoma
TCGA	The cancer genome atlas
TIMER	Tumor immune estimation resource
TNBC	Triple-negative breast cancer
TPM	Transcripts per million
Treg	Regulatory T cell
UPS	Undifferentiated pleomorphic sarcoma
WDLPS	Well-differentiated liposarcoma
